# Evaluating the online impact of reporting guidelines for randomised trial reports and protocols: a cross-sectional web-based data analysis of CONSORT and SPIRIT initiatives

**DOI:** 10.1007/s11192-022-04542-z

**Published:** 2022-10-17

**Authors:** Enrique Orduña-Malea, Adolfo Alonso-Arroyo, José-Antonio Ontalba-Ruipérez, Ferrán Catalá-López

**Affiliations:** 1 Department of Audiovisual Communication, Documentation and History of Art, Universitat Politècnica de València, Valencia, Spain; 2 Department of History of Science and Documentation, University of Valencia, Valencia, Spain; 3 Joint Research Unit CSIC–University of Valencia, UISYS, Valencia, Spain; 4 Department of Health Planning and Economics, National School of Public Health, Institute of Health Carlos III, Madrid, Spain; 5 Department of Medicine, University of Valencia/INCLIVA Health Research Institute and CIBERSAM, Valencia, Spain; 6 Knowledge Synthesis Group, Clinical Epidemiology Program, Ottawa Hospital Research Institute, Ottawa, Canada

**Keywords:** Clinical trials, Reporting guidelines, Altmetrics, Article-level metrics, Webometrics, Link analysis, CONSORT, SPIRIT, Scientific impact, Online impact

## Abstract

Reporting guidelines are tools to help improve the transparency, completeness, and clarity of published articles in health research. Specifically, the CONSORT (Consolidated Standards of Reporting Trials) and SPIRIT (Standard Protocol Items: Recommendations for Interventional Trials) statements provide evidence-based guidance on what to include in randomised trial articles and protocols to guarantee the efficacy of interventions. These guidelines are subsequently described and discussed in journal articles and used to produce checklists. Determining the online impact (i.e., number and type of links received) of these articles can provide insights into the dissemination of reporting guidelines in broader environments (web-at-large) than simply that of the scientific publications that cite them. To address the technical limitations of link analysis, here the Debug-Validate-Access-Find (DVAF) method is designed and implemented to measure different facets of the guidelines’ online impact. A total of 65 articles related to 38 reporting guidelines are taken as a baseline, providing 240,128 URL citations, which are then refined, analysed, and categorised using the DVAF method. A total of 15,582 links to journal articles related to the CONSORT and SPIRIT initiatives were identified. CONSORT 2010 and SPIRIT 2013 were the reporting guidelines that received most links (URL citations) from other online objects (5328 and 2190, respectively). Overall, the online impact obtained is scattered (URL citations are received by different article URL IDs, mainly from link-based DOIs), narrow (limited number of linking domain names, half of articles are linked from fewer than 29 domain names), concentrated (links come from just a few academic publishers, around 60% from publishers), non-reputed (84% of links come from dubious websites and fake domain names) and highly decayed (89% of linking domain names were not accessible at the time of the analysis). In light of these results, it is concluded that the online impact of these guidelines could be improved, and a set of recommendations are proposed to this end.

**Supplementary Information:**

The online version contains supplementary material available at 10.1007/s11192-022-04542-z.

## Introduction

Scientific publications are essential output of any research work, constituting the primary channel for the dissemination of methods and results with the immediate research community, but also, ultimately, with the whole of society. The reliability and relevance of this research is, moreover, essentially guaranteed by the publication of the corresponding article.

In health and medical research, there is evidence, however, that published articles are often poorly reported (Glasziou et al., 2014; Vinkers et al., 2021). Inadequate reporting can be problematic as it can mean that misleading results and biased conclusions are used by healthcare providers, patients, and their families. In response, reporting guidelines have been developed to help ensure the transparency, completeness, and clarity of published articles. Most such guidelines consist of a checklist or explicit recommendations for authors (though also for peer reviewers and journal editors) as to what information should be included when reporting a specific type of study (Moher et al., 2010a, 2014). Most of these reporting guidelines are published in scientific peer-reviewed journals to better promote and disseminate the guideline recommendations to the scientific community. In some occasions, the same reporting guideline can be published by different journals at the same time.

The number of randomised trial related articles published in prominent medical journals has increased considerably in recent decades (Catalá-López et al., 2020). Randomised trials, when appropriately conducted and reported, can provide the most reliable information on the efficacy of interventions for informing healthcare decision-making. In this regard, the Consolidated Standards of Reporting Trials (CONSORT) and Standard Protocol Items: Recommendations for Interventional Trials (SPIRIT) statements provide evidence-based guidance to report essential methodological components on what should be included by those preparing and reviewing articles and protocols of randomised trials, respectively. The CONSORT statement was first published in 1996 and updated twice (Begg et al., 1996; Moher et al., 2001; Schulz et al., 2010a). It includes a 25-item checklist (and flow diagram) for reporting how a parallel-group randomised trial was designed, conducted, and interpreted. Multiple extensions of the CONSORT statement have been published to provide additional guidance for randomised trials with more specific designs (e.g., adaptive trials, cluster trials, pilot, and feasibility studies), data (e.g., equity, harms, and patient reported outcomes), and interventions (e.g., non-pharmacological, social and psychological, and interventions involving artificial intelligence). Similarly, the SPIRIT statement was published in 2013 and provides a 33-item checklist for clinical trial protocols (Chan et al., 2013a). Important details for each checklist item can be found in the explanatory (“Explanation and Elaboration”) papers, which outline the principles underpinning the guidelines and provide published examples of complete and transparent reporting (Chan et al., 2013b; Moher et al., 2010b). The CONSORT and SPIRIT statements form part of a broader international initiative to improve the reporting of health research, the so-called “Enhancing the QUAlity and Transparency Of health Research (EQUATOR) Network” (Altman & Simera, 2016).

### Reporting guidelines in health research from meta-research approach

The CONSORT statement is perhaps the most important reporting guideline for health research, the development of which many other reporting standards are based. It has been recognised among the major milestones in health research methods of the twentieth century (Gabriel & Normand, 2012). The impact and uptake of CONSORT has been measured using several metrics. For example, since its original publication in 1996, the main CONSORT publications have received more than 12,000 citations by other research articles (Shamseer et al., 2016; Caulley et al., 2020a). It is currently known that the CONSORT statement has been endorsed by over 600 health and medical journals, but also by major editorial organizations including the International Committee of Medical Journal Editors (ICMJE) and the World Association of Medical Editors (WAME). Journal endorsement of CONSORT, SPIRIT and other reporting guidelines typically occurs in the form of a supportive statement in a journal’s Instructions to Authors.

Previous meta-research studies have analysed the reporting guidelines, covering the scientific collaboration between their developers (Catalá-López et al., 2019), the guidelines’ citation metrics (Caulley et al., 2020a), their appropriateness of use (Caulley et al., 2020b), and their endorsement by high Impact Factor medical journals (Altman, 2005; Hopewell et al., 2008; Shamseer et al., 2016).

The relation between journals’ endorsement of reporting guidelines and the completeness of reporting (i.e., how published health research actually uses the reporting guidelines) has also been studied (Stevens et al., 2014; Turner et al., 2012). For example, a systematic review (Turner et al., 2012) assessed the effect of journal’s endorsement of CONSORT on the reporting of randomised trials they publish. The authors found that journal endorsement of CONSORT statement was associated with more completely reported trials, based on assessments of more than 16,000 trials. A more recently published citation analysis (Caulley et al., 2020a) showed that few authors cited the CONSORT statement when reporting the methods and results of randomised trials published in high-impact medical journals, even though there is evidence of its effectiveness.

Potential knowledge-to-practice gaps in the uptake of reporting guidelines of health research may offer opportunities to explore alternative approaches to examine impact, increase visibility and promote the use of available reporting guidelines. Traditionally, bibliometrics has relied on citation analysis to measure impact of reporting guidelines, health research, and clinical practice guidelines (Kryl et al., 2012; Thelwall & Kousha, 2016; Thelwall & Maflahi, 2016; Thelwall et al., 2017). Link analysis provides researchers with supplementary analytical methods for the study of quantitative aspects and impact of electronic (online) resources (Thelwall, 2004). However, no link-based studies have been conducted to date to examine the online impact of major reporting guidelines of health research (such as the CONSORT and SPIRIT statements). The application of these methods should, we contend, provide insights into the use of reporting guidelines in broader environments than that specifically of the scientific literature which cites them and thus shed light on how they are disseminated.

## Link analysis of online research objects

Hyperlinks are connections from one online object (e.g., HTML documents, entire websites, images, media, textual files, and software) to another. They have two ends or anchors and a direction,^1^ that is, from object A to object B. Each hyperlink originates at the “source” object and points to the “target” object by embedding the target object’s URL in the source object.^2^ This way, users can navigate to these hyperlinked objects (and visit them in a browser or download them, for example), thus improving their navigational experience, facilitating technical interoperability, and enhancing the visibility and findability of these online objects. When these objects are academic publications, each link received by the target object is known as a URL citation (Kousha, 2019).

The statistical analysis of hyperlinks (i.e., link analysis) is an essential method for monitoring online activity. Search engines, for example, use links to build algorithms to rank online objects for a particular user query. In the field of the Information Sciences, this method is used to understand the types of links generated in academic web environments (Bar-Ilan, 2005), to create networks of actors and information resources (Thelwall & Kousha, 2015; Thelwall, 2004), and to determine the impact of online objects (i.e., the number of links received). This work focuses on determining the impact of online research objects, which in this case correspond to articles describing reporting guidelines.

While raw links counts are informative, these counts can be misleading due to the dynamic nature of the Internet (Thelwall, 2006). By including different impact facets, the accuracy and understanding of link counts can be improved. The most important impact facets are summarized as follows.

### Scattering

This facet reflects the fact that an online object can be published on different locations, and therefore using different URL IDs (e.g., journal website, institutional repository, online academic profile, personal website, DOI, etc.) and different formats (e.g., PDF version and HTML version). This way, the total impact (number of links received by a particular object) is scattered through all the different existing URLs referring to the same object (Orduna-Malea & Alonso-Arroyo, 2017).^3^ Therefore, we cannot measure the impact of the object accurately only by considering one of the existing URLs.

### Degree of similarity

This facet reflects the fact that links counts might be correlated with other metrics (e.g., the number of links received by an online object might be correlated with the number of citations received by that object). When a significant positive/negative correlation is found, the strength of the relationship between the metrics measured is high, and consequently, we can use the value of one variable to predict the value of the other variable.

### Broadness

This facet measures the number of different domain names linking to one specific online object. This way, an object increases its broadness as the linking domain names counts increase. A webpage can generate links towards one online object massively, distorting the value of links counts. However, by counting linking domain names instead of linking webpages we can figure out the impact more accurately (Orduna-Malea & Alonso-Arroyo, 2017).

### Diversity (entity level)

Considering the entities responsible of linking domain names, this facet measures how many different types of entities generate links to one object. For example, we can break down entities into universities, media, companies, etc. Attaining links from a low number of entity types means specialization, whereas the opposite reflects diversity. This concept is referred to in this work for the first time.

### Diversity (genre level)

This facet measures how many different object genres generate links to one online object. For example, we can break down object genres into journal articles, book chapters, news posts, encyclopaedia entries, tweets, etc. While attaining links from a low number of object genres means specialization, the opposite reflects diversity. This concept is referred to in this work for the first time.

### Reputation

This facet complements the impact broadness by measuring the number of links received from reputable domain names. In this work, reputation has been operationalized by the Trust Flow indicator, provided by the Majestic link intelligence tool. A website’s Trust Flow value increases when it receives links from reputable websites, which in turn are those with higher Trust Flow values. This recursive algorithm starts operating through a curated list of websites manually categorized as reputable by a community of experts (Jones, 2012). As fake or low-quality domain names can be created to link online objects to inflate their links counts, by measuring links from reputed domain names we can measure impact more accurately. This measure has been previously successfully applied in other link-based studies (Orduña-Malea, 2021; Orduña-Malea & Costas, 2021).

### URL decay

This facet measures the loss of links (and consequently the loss of impact) pointing to an online object over time. This is a consequence of the change or disappearance of the linking online objects (Koehler, 1999; Oguz & Koehler, 2016; Payne & Thelwall, 2007). This issue produces remarkable effects on the academic web (Kumar & Kumar, 2012; Spinellis, 2003; Yang et al., 2012). Consequently, measuring only current links can show misleading results when total online impact is required.

Link analysis has been applied to the study of a range of different agents engaged in the field of science and technology via the measurement of specific aggregate online objects, including academic journal websites (Vaughan & Thelwall, 2003), scientific software websites (Orduña-Malea & Costas, 2021), authors’ personal websites (Barjak et al., 2007), patents (Orduña‐Malea et al., 2017; Font-Julián et al., 2022), open access repositories (Aguillo et al., 2010), research groups (Barjak & Thelwall, 2008), research projects (Dudek et al., 2021), university websites (Ortega & Aguillo, 2009; Thelwall & Zuccala, 2008), and public health entities (Ontalba-Ruipérez et al., 2016).

However, the application of link analysis at the article-level remains scarce for two main reasons. First, commercial search engines – Google included – eliminated their search facilities (Thelwall, 2021), thus limiting the use of massive link data, and requiring the employment of alternative link data sources that are not designed to analyse research online objects, such as scientific publications, quantitatively. Second, although hyperlinks can be used to obtain supplementary evidence of the wider impact of academic research, “link spam is widespread, and hyperlinks can be generated automatically in large numbers for legitimate reasons” (Thelwall & Kousha, 2015). This means that link counts fail to capture the wider impact of research unless a data cleansing process is employed.

Against this backdrop, this study seeks to determine the impact of a set of reporting guidelines for randomised trial related articles by means of link analysis. To do so, a tailored data process (the DVAF method) is developed to address the limitations of raw link analysis, based on the use of the link intelligence tool Majestic as a data source.

### Objectives

The purpose of this study is two-fold: (1) to determine the online impact and dissemination of CONSORT and SPIRIT statement related articles; and (2) to develop a link analysis method that can increase the accuracy of online impact studies. To this end, the following research questions are posed:

RQ1a.: How many URL citations do the reporting guidelines receive?
RQ1b.: Through which URL IDs are they most linked?
RQ2.: Do URL citations to reporting guidelines correlate with citation and alternative metrics indicators?
RQ3.: Do URL citations to reporting guidelines come from a wide number of websites?
RQ4.: Do URL citations to reporting guidelines come from a wide number of entity types and object genres?
RQ5.: Do URL citations to reporting guidelines come from reputed websites?
RQ6.: Do URL citations to reporting guidelines decay over time?

## Methods

We conducted a cross-sectional analysis of link-based data for the CONSORT and SPIRIT initiatives. All journal articles related to the CONSORT and SPIRIT statements were identified by a senior scientist (FC-L). To do so, the CONSORT,^4^ SPIRIT^5^ and EQUATOR^6^ Network websites were inspected manually (last search dated 2 October 2021), as these resources report the journal articles in which the main reporting guidelines and their extensions have been officially published. For the present study, we included articles published in English concerning the main CONSORT and SPIRIT statements, and their related extensions for trial reports and protocols. Translations of any of the articles into other non-English languages (e.g., CONSORT translations into Chinese or Spanish) were excluded. Editorials, book chapters, and corrections were likewise excluded. This process yielded a total of 38 reporting guidelines described in 65 articles. The supplementary material (Appendix A) lists the reporting guidelines collected and reports the articles in which each guideline is described.

### Webometric analysis

The URLs related to each of the articles included (henceforth, referred to as the target URLs) were located. To do so, the DOI, the journal website of the article, the PubMed ID, and the PMC ID (when available) were considered for each article. While other URL IDs may exist, these four were deemed sufficient to comply with the objectives of this study. All the URLs located are listed in the raw data supplementary file (*Target URLs tab*). This process yielded 222 URLs.

Having identified the target URLs, the source URLs (i.e., the URLs of those online objects linking to at least one target object) were then located. The source URLs were identified using the historic index of Majestic,^7^ a professional link intelligence tool.

The Majestic tool has been previously used and tested for link-based studies (Orduña-Malea, 2021; Orduña-Malea & Aguillo, 2022; Orduña-Malea & Costas, 2021). This tool is specifically dedicated to carry out professional link-based analyses, offering the following advantages: (a) availability of a wide range of basic (e.g., number of links received) and composed (e.g., Trust Flow) indicators; (b) availability of quantitative (e.g., number of linking domain names) and qualitative (e.g., language of the linking source object) indicators; (c) availability of four different analysis levels (URL, subfolder, subdomain and domain); and d) availability of two complementary databases (the fresh index, covering links crawled during the last four months; and the historic index, covering all links generated since 2006, including both active and deleted links). Specifically, the use of the Trust Flow indicator has been proved to be an effective method to filter out those websites attaining a huge quantity of links from dubious sites, improving the accuracy of link analysis (Orduña-Malea, 2021; Orduña-Malea & Aguillo, 2022).

Majestic includes a self-made search engine through which crawls the entire Web, indexing all URLs found. For each URL indexed, Majestic automatically calculates a wide range of link-based metrics.^8^ The historic index includes all URLs found since June 2006, covering more than 3800 billion unique URLs as of July 2022.

For this study, the Majestic’s internal online search feature was used, setting up the historic index and the URL-level analysis. Each target URL was inserted as a search term, and the database returned all the available metrics related to those URLs, including all linking source URLs. As the dataset is limited (65 articles and 222 URLs), this process was carried out manually by a senior researcher (EO-M).

All source URLs were directly downloaded in CSV file format. In addition, for each source URL, a pair of online impact-related flow metrics (Citation Flow and Trust Flow) was gathered (see Table 1 for description), along with other technical and descriptive link-based indicators (see the *Source URLs tab* in the raw data supplementary file). This procedure yielded a total of 240,128 hyperlinks from 204,993 different objects.

**Fig. 1:**
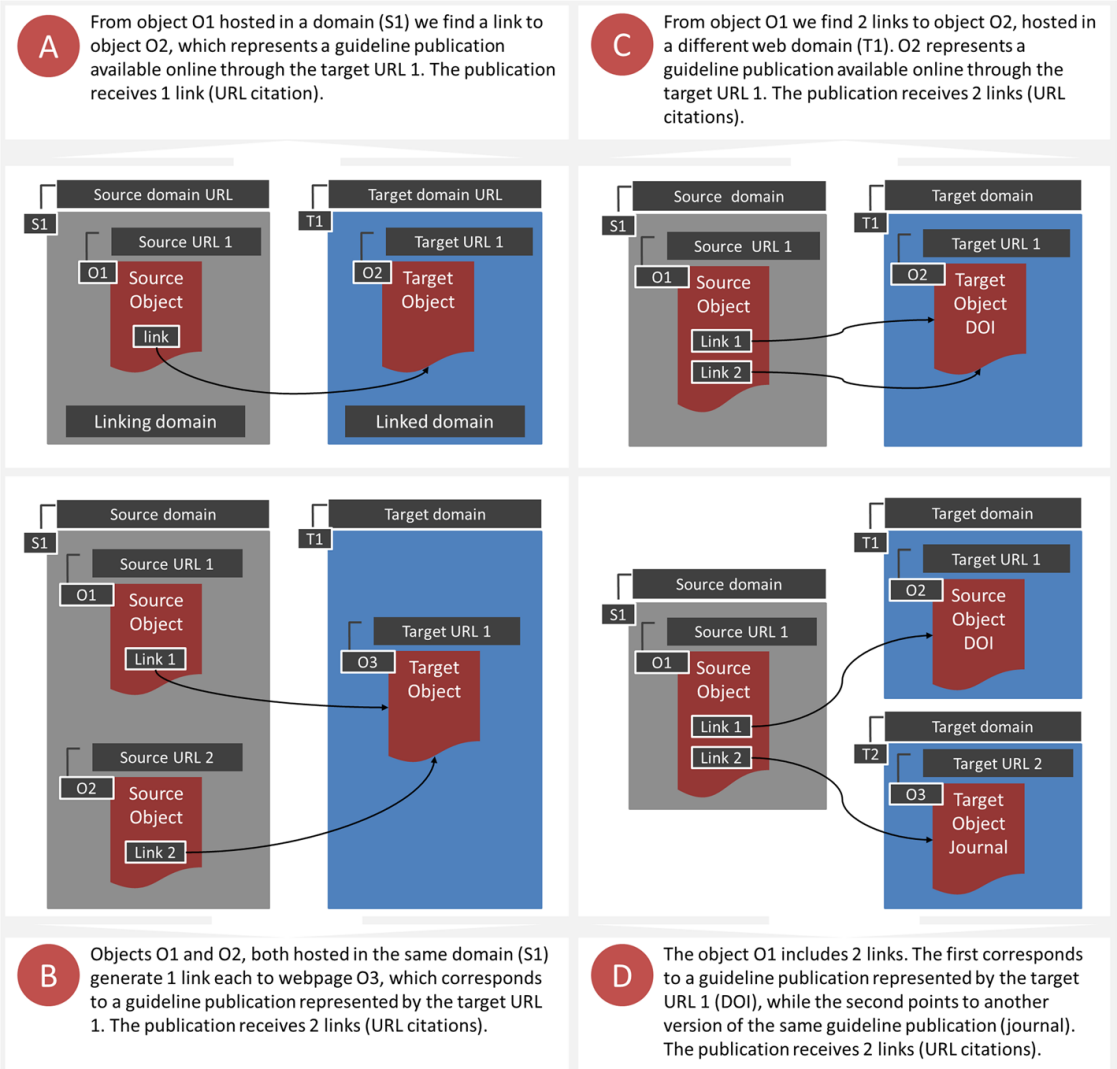
Hyperlink connections between source and target objects: the single case (**A**), the multiple source case (**B**), the multilink source case (**C**) and the multiple target case (**D**)

The hyperlink connections established between the source and target objects fall into one of four categories (see Fig. 1 for a summary): The *single case* (Fig. 1A) exemplifies the most common situation, where an article is linked from a single source object; in the *multiple source case* (Fig. 1B) different source objects link to the same article; in the *multilink source case* (Fig. 1C) the same source object provides several links to the same article; and, finally, in the *multiple target case* (Fig. 1D) different articles are linked by the same source object.

Multilink source cases artificially inflate the number of links received by one specific target object. Likewise, multiple source cases can also inflate link counts when source objects are duplicates (e.g., the http and https versions of one object). These limitations are due both to the web dynamics and the way Majestic offers raw link data. For this reason, a filtering process is required. The 204,993 different source URLs were subsequently filtered following a four-step process – debugging, validating, accessing, and finding – or the DVAF method, proposed in this work, and detailed below.

### The DVAF (debugging, validity, accessibility, findability) cleansing process

**Fig. 2:**
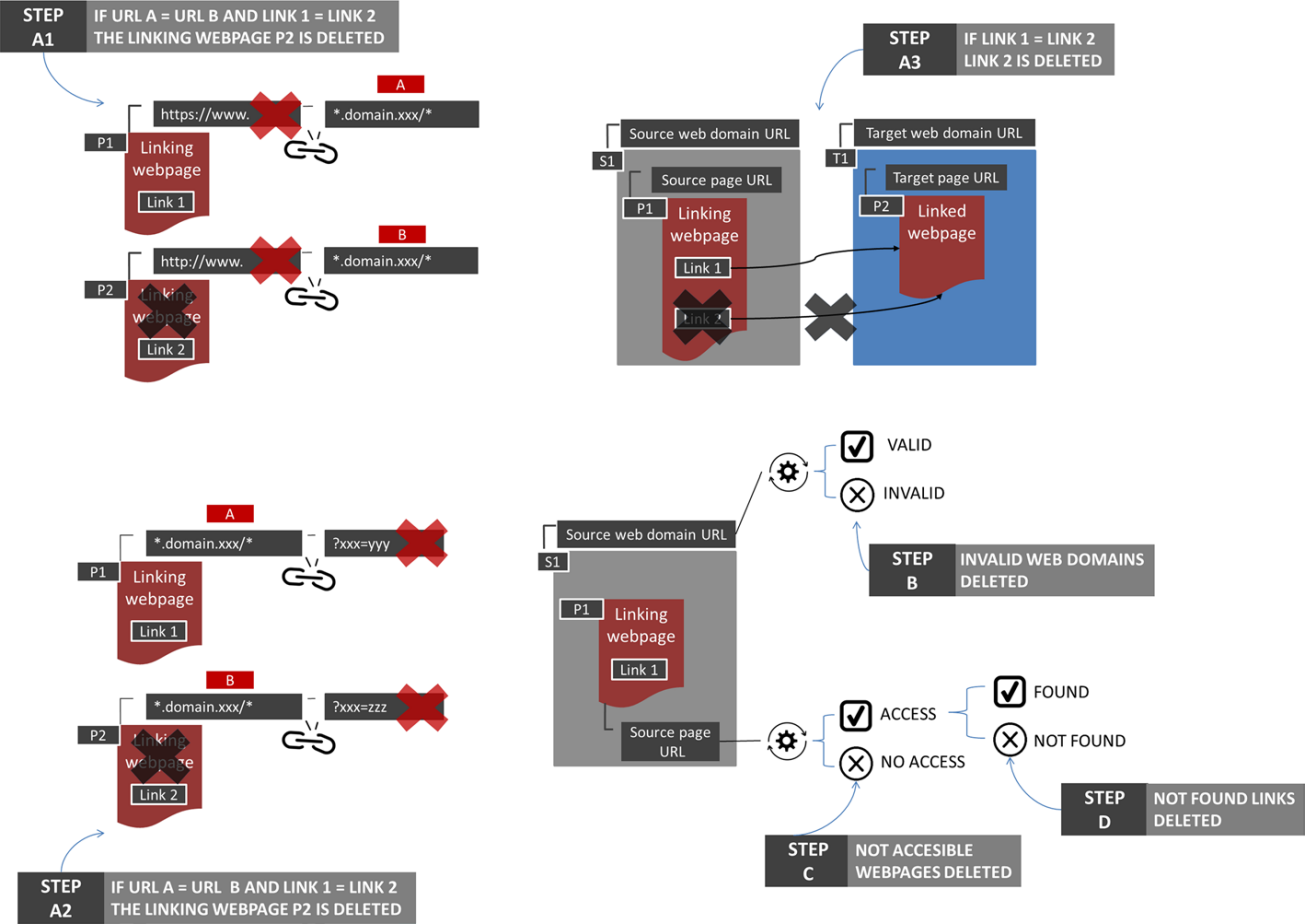
The *DVAF cleansing process*: data debugging (step A), validity (step B), accessibility (step C), and findability processes (step D)

In the first step, the debugging process, URL protocols (e.g., http, https, and www) and URL query parameters (e.g., ‘?ijkey = 221ca3a’ and ‘?utm_source = hs_email’) were extracted from the source URLs (see Fig. 2, steps A1 and A2). Each duplicated pair ‘Source URL’ – ‘Target URL’ was then removed, as were all duplicated links embedded in the same source object (see Fig. 2, step A3).

In the second step, the validation process, each website referred to by each source URL was accessed manually. Here, all forwarded URLs (those URLs automatically redirecting to other URLs), obsolete URLs (those URLs taking the user to inaccessible websites), and dubious URLs (e.g., URLs related to websites offering illegal products or sexual content) were excluded (see Fig. 2, step B).

Entities responsible for the websites were subsequently typified, such as higher education institutions (e.g., ox.ac.uk, northwestern.edu), companies (e.g., nursingresearchwriters.net, orange.com), organizations (e.g., ons.org, rand.org), publishers (e.g., frontiersin.org, biomedcentral.com), personal websites (e.g., arasharya.de, callinanllc.com), etc.

To do this, a bottom-up process (see Orduña-Malea, 2021) was conducted by a senior researcher (EO-M), who manually accessed each website, browsed through the website contents and “about” sections, and determined the entity type. After a first iteration, a draft of categories was set, which was discussed and agreed by the research team, generating a final categorization scheme of 31 entity types. Then, a second iteration was carried out to fix errors. The Appendix D (source entity types) includes the list of all categories along with a brief description.

The third step involved carrying out an accessibility task. Because a source online object can be deleted while the domain name’s URL remains active, a python script was written to obtain the HTML response status code^9^ of each of the source URLs from the validated domain names in order to confirm their accessibility. All online objects with no access were deleted (see Fig. 2, step C). Data related to all HTML responses can be consulted in the raw data supplementary file (*html response code tab*).

The fourth step involved determining the genre of the specific objects within each source domain name linking to reporting guidelines. First, each source object was manually checked to verify whether the link was still active, and if so, to typify the object genre. This process was manually processed by two senior information specialists (AA-A and J-AO-R). Second, if the link was found on the source online object, the genre was noted down (e.g., academic article, encyclopaedia entry, personal webpage, etc.). To typify each object, a bottom-up process was followed. A first iteration created a basic set of genres, which was subsequently agreed by the authors by merging and polishing a final scheme of 21 genres. Then, a second iteration was carried out to assign each source object with a specific genre. Third, each link was also associated with a potential purpose. Links embedded in publications (journal articles, books, theses, etc.) were associated to scientific purposes; links embedded in news, informative posts or encyclopaedia entries were associated to informational purposes; links embedded on author personal websites or list of references created by research groups, research centres or libraries were associated to informative purposes. Finally, links embedded in bibliographic records (such as the automatic page created by a repository which describes an article and includes a link to that paper) were associated to a functional purpose. The Appendix F includes all the genres created, a brief scope, and an illustrative example of each of the genres found.

After debugging and validating, the results include all URL citations that at one time existed, regardless of whether they remain active today. These data are used to show the online impact achieved by the reporting guidelines. Data obtained after carrying out the accessibility task are used to check the URL decay of these source URLs.

Gephi^10^ v. 0.9.1 was used to generate a link analysis map connecting the source URLs to the target URLs.

### Bibliometric analysis

For each article, the following reporting guideline descriptive fields were considered: reporting guideline name, parent guideline (e.g., CONSORT, SPIRIT, and CONSORT/SPIRIT), extension (yes/no), study design (e.g., clinical trials, experimental studies, study protocols), and application (e.g., intervention, outcomes, whole report, protocol, and abstract). All data can be consulted in the raw data supplementary file (*Guidelines tab*). Scopus was then used to gather the following bibliographic metadata for each publication: journal name, year of publication, DOI, document type (article, review, editorial), authorship, affiliation, keywords, citations received, references cited, and funding information (see the *Publications tab* in the raw data supplementary file). Articles G6-P11 and G10-P02 were not found in Scopus and were, therefore, eliminated from the analysis.

### Altmetric analysis

The *PlumX analytics* data provider^11^ was selected on the grounds that it is linked to the Scopus database. *Plum Analytics* is a subscription-based platform founded in 2012 which provides a wide range of Altmetrics (categorized into citations, usage, captures, mentions, and social media metrics) for each publication (Williams, 2019). The literature has pointed out PlumX as one of the major altmetrics data providers, especially to capture Mendeley readership (Ortega, 2018; Zahedi & Costas, 2018; Ortega, 2020; Karmakar et al., 2021).

Additionally, *PlumX* captures both clinical and policy citations (Kryl et al., 2012), of interest to capture broader impact of the reporting guidelines analyzed. *PlumX* covers the clinical guidelines indexed in *PubMed*, *Dynamed Plus topics*, and NICE (*National Institute for Health and Care Excellence*), and those policy documents indexed in the *Overton* database (Fang et al., 2020; Szomszor & Adie, 2022). The user can access the *PlumX* clinical citations cards^12^ and policy citations card^13^ to identify the specific citing documents and check whether the citations have been located correctly.

To obtain the alternative metrics related to each of the reporting guidelines under study, the DOI of each article was used as a data seed, thus furnishing a wide range of altmetrics for each reporting guideline (see raw data supplementary file, *PlumX tab*) through the API service.^14^

Data prevalence (i.e., publications with data collected for one specific metric) was scarce for a number of metrics (e.g., 73.4% of the articles received no mentions from blogs). To avoid this issue, only those metrics with a data prevalence of at least 50% (i.e., half the publications presented data) were included. As a result, eventually citations from clinical guidelines and policy documents, Mendeley readers, Tweet counts, Facebook counts, abstract views, and export saves were the metrics considered.

Data related to all three analytic techniques (link analysis, Bibliometrics and Altmetrics) were collected and statistically analysed as of 14 October 2021. The main metrics collected and measured in this study are summarized and described in Table 1.

**Table 1:** Metrics measured for each guideline publication

Metric	Source	Scope
Citation counts	Scopus	Number of citations received by each article from other publications indexed in Scopus
Clinical citations	PlumX	Includes the number of clinical guidelines from PubMed that reference the guideline article; the number of Dynamed Plus Topics that reference the guideline article, and the number of clinical guidelines from NICE that reference the guideline article
Policy citations	PlumX	Number of policy documents that reference each guideline article, from Overton
Abstract views	PlumX	Number of times the abstract of the guideline article has been viewed*
Export saves	PlumX	Number of times a guideline article’s citation has been exported directly to bibliographic management tools or as file downloads, and number of times a guideline articles’ citation/abstract and HTML full text (if available) have been saved, emailed or printed
Reader counts	PlumX	Number of people who have added the guideline article to their Mendeley library
Facebook counts	PlumX	Number of times the target URL was shared, liked, or commented on
Twitter counts	PlumX	Number of tweets and retweets that mention the target URL
Source online resource counts	Majestic	Number of source resources mentioning a target object
Source domain name counts	Majestic	Number of source domain names which include at least one online object linking to a target object
URL citations	Majestic	Total number of links received by an article from source online objects
Citation Flow	Majestic	Score on a scale between 0–100 achieved by one website, based on the number of hyperlinks it receives. It measures how often a URL is linked (Jones, 2012) and, so, it measures the number of links received
Trust Flow	Majestic	Score on a scale between 0–100 achieved by one URL, based on the number of hyperlinks (and clicks on these links) from trusted seed sites that the URL receives. As such, it measures authority and ability to generate web traffic (Jones, 2012)
Scattering breadth	Majestic	Number of different URLs representing the same online object. Note, in this study, this value is fixed at 4
Impact breadth	Majestic	Number of different source domain names linking to one article
Reputation breadth	Majestic	Number of source domain names referring to one specific online object achieving Trust Flow and Citation Flow values equal to or higher than 50
Diversity breadth (entity level)	Majestic	Number of different entities responsible for websites from which an online object receives links
Diversity breadth (object level)	Majestic	Number of different objects genre from which an online object receives links

*Including Airiti iRead eBooks, Airiti Library, CABI, Digital Commons, DSpace, EBSCO (historical only), ePrints, Expert Gallery Suite, RePEc, SciELO, and SSRN

To analyse the degree of similarity between article-level metrics, the rho-Spearman correlation (alpha-value of 0.01) was applied because of the skewed distribution of the metrics. All statistical analyses were performed with the free version of XLStat.

**Fig. 3:**
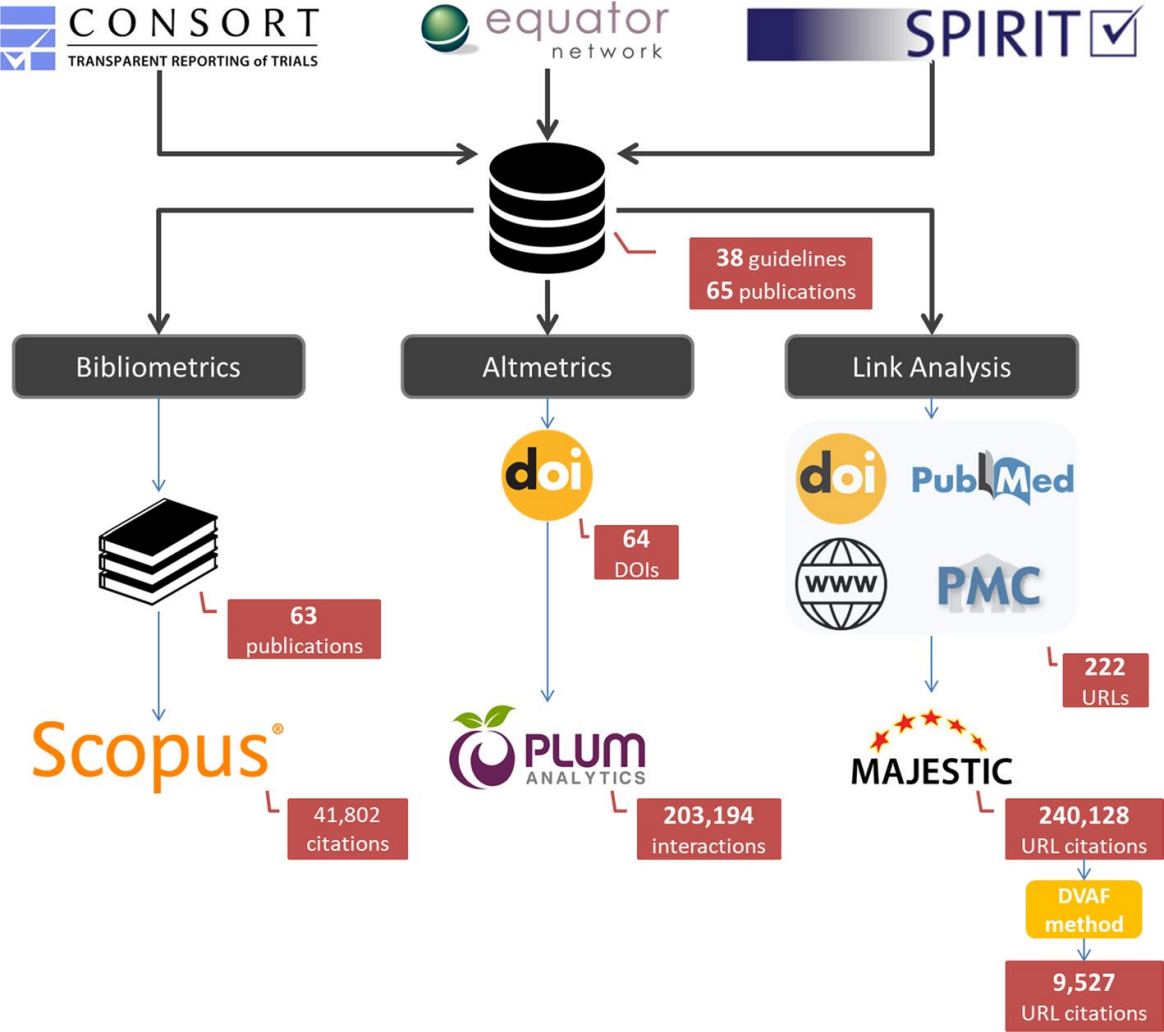
Data gathering process: bibliometric, altmetric and link analysis methods

The whole data collection process is summarized in Fig. 3.

## Results

### URL citations to reporting guidelines

A total of 15,582 links to journal articles related to the CONSORT and SPIRIT statements were identified. CONSORT 2010 (described in ten articles) and SPIRIT 2013 (described in two articles) were the reporting guidelines that received most links (URL citations) from other online objects (5328 and 2190, respectively).

If we consider the URL ID linked by source objects, we detect an initial web scattering effect. Source objects mainly linked the DOI URL version of the articles (82.9% of all links created refer to this URL ID), with this URL ID being the most linked in 29 of the 38 guidelines studied (Table 2). The journal URL ID was also commonly used, concentrating 9.6% of all links received, and was the most linked URL ID for a few specific guidelines (including, for example, the CONSORT and SPIRIT extensions for interventional trials involving artificial intelligence, i.e., CONSORT-AI, SPIRIT-AI). The remaining URL IDs presented very few links.

**Table 2:** Number of URL citations received by reporting guidelines according to each URL ID

ID	Reporting guideline	DOI	JOURNAL	PMC	PUBMED	ALL
G06	CONSORT 2010	4,574	213	344	197	5328
G07	SPIRIT 2013	2,105	43	23	19	2190
G34	CONSORT 2001	960	4	0	7	971
G11	TIDIeR	862	64	NA	9	935
G35	CONSORT-Non-pharmacologic treatment 2008	624	9	0	40	673
G16	CONSORT-Pragmatic Trials	467	13	52	27	559
G20	CONSORT-Cluster	474	6	NA	23	503
G32	CONSORT-AI	60	341	0	86	487
G08	CONSORT-Pilot and Feasibility	393	77	0	10	480
G22	STRICTA	400	2	9	4	415
G17	CONSORT-Abstracts	271	13	45	32	361
G12	CONSORT-Harms	259	16	NA	44	319
G21	CONSORT-Non-inferiority	299	5	NA	1	305
G31	SPIRIT-AI	63	177	1	22	263
G13	CONSORT-EHEALTH	143	51	5	2	201
G30	ACE	50	73	9	54	186
G29	CONSORT SW-CRT	85	60	3	1	149
G14	CONSORT-PRO	130	12	NA	1	143
G19	CONSORT-Herbal	125	6	0	7	138
G23	CONSORT-SPI	66	57	3	7	133
G02	SPIRIT-PRO	61	64	NA	2	127
G33	CONSORT 1996	89	NA	NA	19	108
G18	CONSORT-Non-pharmacologic treatment 2017	87	3	NA	2	92
G27	CONSORT crossover	33	35	1	1	70
G37	CONSORT-ROUTINE	8	14	9	27	58
G01	TIDIeR-PHP	36	17	NA	2	55
G04	CONSORT-Within person	45	5	3	1	54
G28	CONSORT multi-arm	18	35	NA	NA	53
G38	CONSERVE	1	41	NA	2	44
G36	TIDieR-Placebo	21	14	1	2	38
G10	CONSORT-CENT	29	8	0	0	37
G03	CONSORT-Equity	19	15	NA	NA	34
G05	CONSORT-CHM 2017	29	NA	NA	1	30
G24	SPIRIT-TCM	15	NA	NA	NA	15
G09	Simulation Research	10	1	NA	2	13
G25	SPENT	7	NA	NA	3	10
G26	CENT for TCM	4	NA	NA	1	5
G15	CONSORT-C	3	NA	NA	NA	3
	Total	12,925	1,494	508	658	15,585
	%	82.9	9.6	3.3	4.2	100

If we disaggregate the data at the article-level (supplementary material, Appendix B), we observe that the use of journal URL versions was higher for more recent publications (i.e., those published in 2020 and 2021). For example, article G32-P1 (CONSORT-AI published in Nature Medicine in 2020) (Liu et al., 2020) obtained seven citations, 28 links to its DOI URL ID, and 246 links to its journal URL ID. Similarly, article G38-P1 (the joint extension for CONSORT and SPIRIT, the so-called CONSERVE statement for clinical trials modified due to the COVID-19 pandemic and extenuating circumstances, published in JAMA in 2021) (Orkin et al., 2021) obtained one citation, one DOI URL link, and 40 journal URL links.

**Fig. 4:**
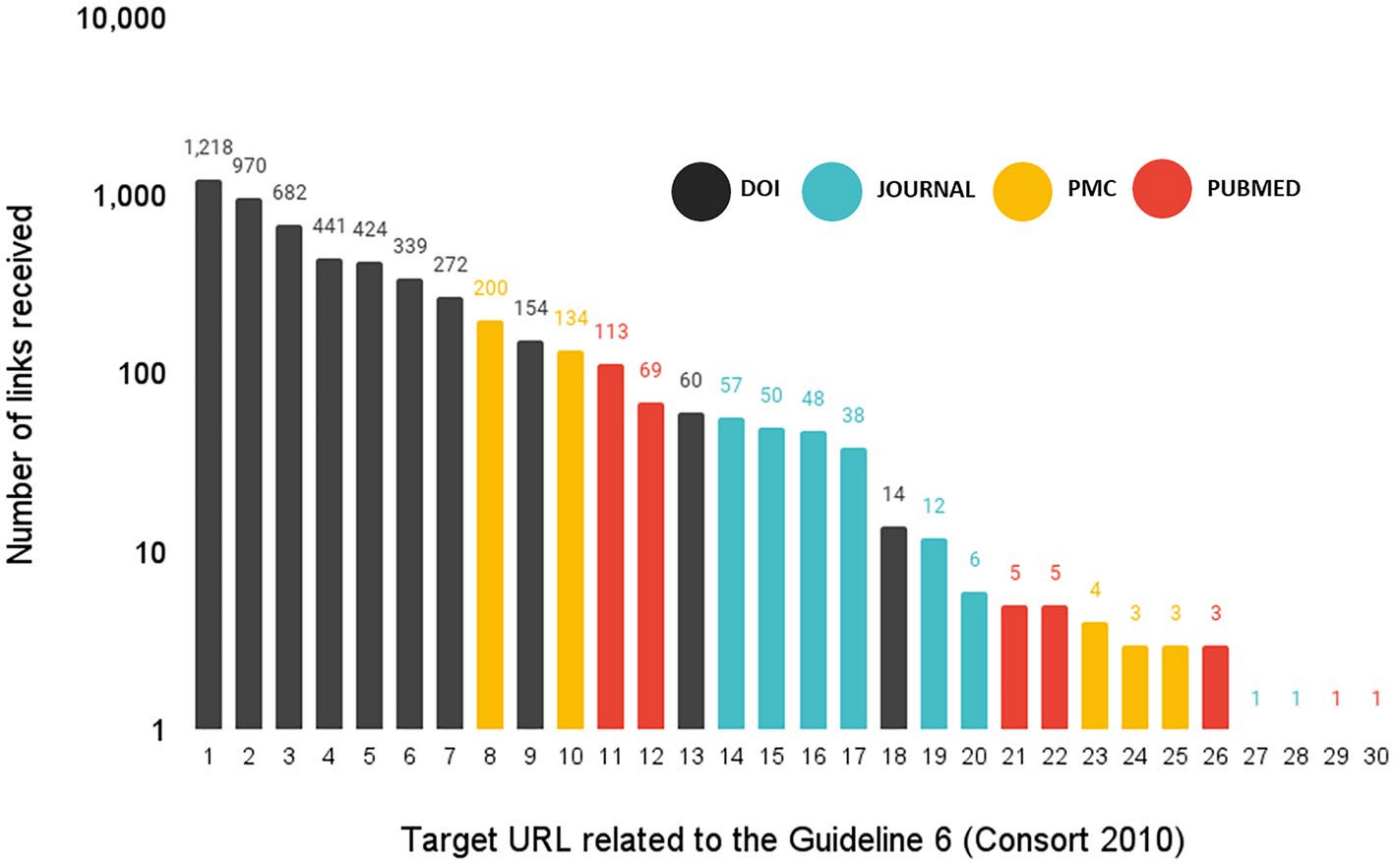
URL citations received by target URLs related to the CONSORT 2010 statement

The analysis conducted at the article-level also revealed a second web scattering effect for those guidelines described in more than one article. For example, the CONSORT 2010 statement receives 5328 links from 30 different URLs, related to the ten articles in which this guideline is described (Fig. 4).

Figure 4 presents a skewed distribution in which DOI URL IDs obtained the greatest number of links. This distribution reflects the unequal impact of each of the articles describing the CONSORT 2010 statement. Article G06-P3 (CONSORT 2010 statement: updated guidelines for reporting parallel group randomised trials, published in the BMJ) received 1581 URL citations (when including all four URL IDs analysed in this study) and article G06-P9 (CONSORT 2010 explanation and elaboration document published in the BMJ) received 1211. However, the remaining articles obtained a smaller number of links.

### Correlation of URL citations to reporting guidelines with citation and alternative metrics

The unequal impact of the articles describing the same guideline was evident not only as regards links received but also when measuring citations and alternative metrics (Table 3). On occasions, the article with the highest impact was the one with the highest Journal Impact Factor (JIF) (e.g., CONSORT extension for Herbal Medicine: G19-P1; STRICTA: G22-P1). However, in other instances, the JIF was not a key driver of impact (e.g., CONSORT 2010: G06-P8; CONSORT extension for Abstracts: G17-P1).

**Table 3:** Article-level impact metrics of those reporting guidelines described in different articles

ID	PUB	JIF	URL Citations	Citations	Clinical citations	Policy citations	Exports saves	Facebook counts	Reader counts	Tweet counts
G06	P1	16.729	456	1,834	16	64	163	0	615	0
	P2	5.750	738	1,696	2	25	37	3	529	6
	P3	13.471	1,581	3,185	15	97	37	10	2583	7
	P4	3.753	155	499	1	19	21	0	292	0
	P5	4.392	62	184	1	2	0	0	300	0
	P6	15.617	433	709	3	37	34	0	539	2
	P7	2.080	334	450	0	8	15	48	239	1
	P8	33.633	14	74	0	3	0	0	104	2
	P9	13.471	1,211	2,618	14	60	17	64	2852	31
	P10	3.753	344	1,080	3	14	32	1	815	2
G07	P1	16.104	1,214	1,851	3	62	58	715	1228	7
	P2	16.378	976	1,347	5	36	15	29	1577	93
G08	P1	N/A	231	270	0	1	7	65	552	58
	P2	20.785	249	391	1	2	0	247	620	107
G10	P1	19.697	24	85	2	4	1	0	124	37
	P2	19.697	13	49	1	2	1	123	105	32
G17	P1	28.409	95	269	13	10	0	0	408	1
	P2	12.185	266	369	16	19	29	2	392	20
G19	P1	14.780	109	399	21	17	31	0	226	0
	P2	2.440	29	136	0	6	7	0	158	0
G22	P1	15.617	195	380	5	10	7	17	231	2
	P2	1.498	23	46	0	1	106	0	44	0
	P3	1.498	130	215	1	3	5	0	286	0
	P4	1.381	67	110	0	1	62	0	59	1
G23	P1	1.975	68	35	0	1	19	30	144	180
	P2	1.975	65	35	0	1	23	36	151	201
G30	P1	39.890	130	3	0	0	0	163	62	237
	P2	2.279	56	2	0	0	0	0	29	62
G31	P1	39.890	80	4	0	1	0	39	78	92
	P2	53.440	79	2	0	1	0	0	130	1
	P3	24.519	104	2	0	0	0	22	105	120
G32	P1	53.440	305	7	0	1	0	89	159	3
	P2	39.890	97	2	0	1	0	235	89	98
	P3	24.519	85	2	0	0	0	12	101	45
G34	P1	11.130	137	894	13	22	43	0	205	0
	P2	17.569	275	1,854	32	94	28	0	171	0
	P3	13.251	559	2,830	0	108	0	0	308	0
G35	P1	17.457	75	353	5	12	75	0	368	0
	P2	17.457	598	1,514	13	42	136	1	1,012	0

**Fig. 5:**
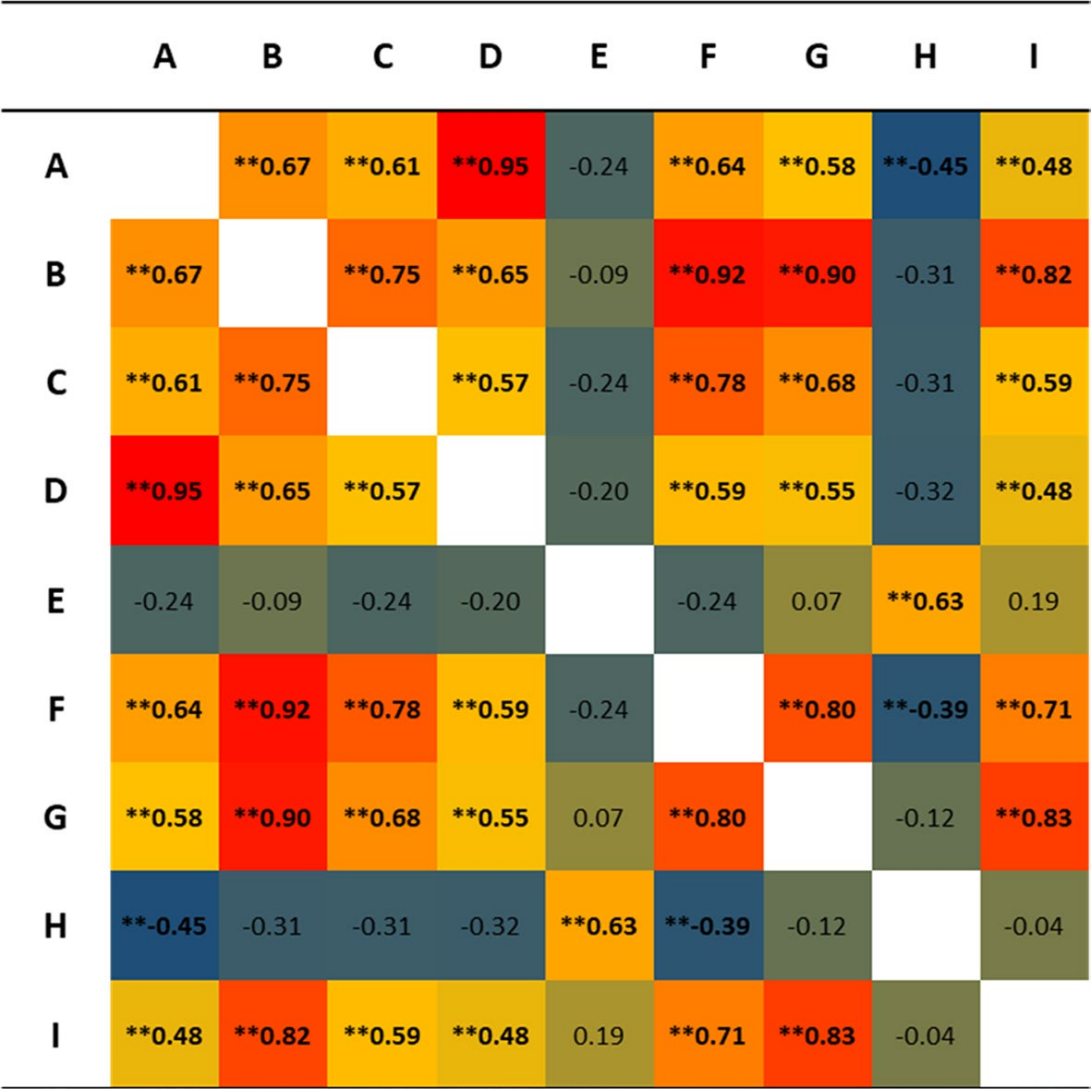
Correlation matrix (Spearman) of URL citations, citations and Altmetrics. **Values that are different from 0 with a significance alpha-value of 0.01. A = Abstract views; B = Citations; C = Clinical citations; D = Exports saves; E = Facebook counts; F = Policy citations; G = Reader count; H = Tweet count; I = Links

The number of URL citations received per article is strongly correlated with the remaining impact-based indicators (Fig. 5), especially with the number of citations (Spearman R = 0.82; p-value < 0.0001; alpha > 0.01) and the number of Mendeley readers (Spearman R = 0.83; p-value < 0.0001; alpha > 0.01). However, no correlation was found with Twitter (Spearman R = −0.04; p-value = 0.753; alpha > 0.01) or Facebook (Spearman R = 0.19; p-value: 0.138; alpha > 0.01), outcomes that present a statistically significant correlation with each other (Spearman R = 0.63; p-value < 0.0001; alpha > 0.01). All the alternative metrics collected can be consulted in the supplementary material (Appendix C).

### Domains linking to reporting guidelines

The number of different domain names linking to the articles (i.e., the impact breath of each article) was limited. The average value is 34.64 linking domain names (median value of 28.5; 90th percentile value of 58). The distribution of the impact breath was skewed, with only three articles exceeding 100 different linking domain names (CONSORT 2010: G6-P3, G6-P9, and CONSORT-AI: G32-P1).

**Fig. 6:**
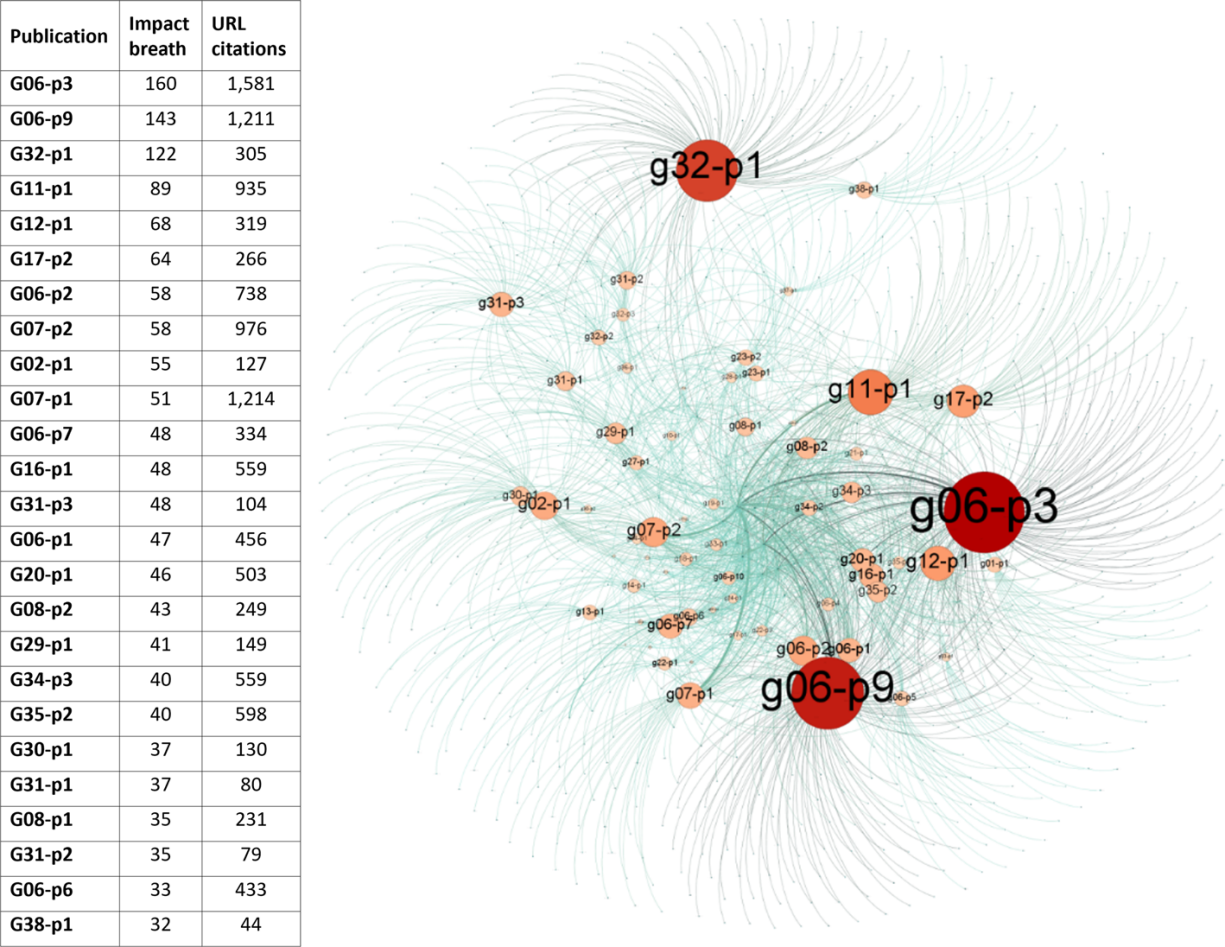
Link connection between articles and linking domain names. Article-domain network powered with Gephi (https://gephi.org). Force-directed layout: Fruchterman-Reingold. Red node: articles; Green nodes: linking domain names; red node size: number of links received; green node size: fixed to 1 to simplify the network lay-out

The number of URL citations generated by each of the linking domain names differed significantly according to each article (Fig. 6, left). Here, the anomalous behaviour of a few articles was evident, the case of CONSORT-AI (G32-P1 had 2.5 URL citations per domain name; very low percentage) and SPIRIT 2013 (G7-P1 had 23.8 URL citations per domain name; very high percentage). Other than these exceptions, the number of URL citations received per domain name was low (average of 5.73).

On the other hand, the linking domain names only linked to a few articles each (average of 2.5; median value of 1), generating a raked visual effect in the article-to-domain network generated (Fig. 6, right). A number of publishers, most notably BioMed Central (with URL citations to 58 articles), Springer (48), PLoS (44), and Frontiers in (41), constituted exceptions, hosting numerous citing publications (for more details, see Table 5).

### Entities and objects linking to reporting guidelines

A total of 38 entity types (diversity breadth) were identified (Table 4), ranging from academic-oriented websites (e.g., publishers, universities, databases, research groups, research centres, academic personal websites, bibliographic databases) to general information-oriented websites (e.g., news, encyclopaedias, information portals), health information-oriented websites (e.g., health organizations, health information portals, health official bodies, hospitals), and commercial-oriented websites (e.g., private companies, shopping). A brief description of the scope of each entity type can be consulted in the supplementary material (Appendix D).

**Table 4:** Domain names and URL citations according to entity type

Entity type	Domain names count	%	URL citations count	%	Avg. TF	Avg. CF
Publisher	192	21.8	10,004	64.2	66.14	62.54
Organization	88	10.0	418	2.7	35.35	40.34
Company	82	9.3	560	3.6	20.57	27.36
Higher Education Institution	74	8.4	2283	14.6	58.03	49.91
Health information hub	65	7.4	321	2.1	15.08	28.16
News Hub	62	7.0	138	0.9	12.56	28.60
Other information hubs	51	5.8	195	1.3	14.93	23.16
Academic database	50	5.7	338	2.2	30.54	39.05
Encyclopaedia	44	5.0	771	4.9	44.50	52.11
Directory	36	4.1	72	0.5	3.88	32.69
Personal website	24	2.7	59	0.4	6.03	13.42
Non-Academic Database	20	2.3	55	0.4	7.00	33.13
App	13	1.5	71	0.5	20.10	31.30
Health government body	11	1.2	100	0.6	81.02	77.94
Science Hub	8	0.9	12	0.1	30.25	37.08
Research centre	7	0.8	11	0.1	20.36	21.36
Online fora	6	0.7	10	0.1	25.20	31.40
Research group	5	0.6	16	0.1	4.19	12.31
Shopping	5	0.6	17	0.1	11.18	24.47
Hospital	5	0.6	5	0.0	26.00	29.60
Blogs provider	5	0.6	17	0.1	79.06	84.06
Tourism	4	0.5	20	0.1	1.75	15.70
Search engine	3	0.3	12	0.1	1.50	6.83
Research project	3	0.3	4	0.0	16.25	18.25
Research institute	3	0.3	25	0.2	25.40	37.48
Clinic	3	0.3	13	0.1	2.46	31.69
Event	2	0.2	6	0.0	9.33	19.00
Videos Hub	2	0.2	18	0.1	24.00	40.22
Images Hub	2	0.2	2	0.0	12.50	28.50
Promos	2	0.2	2	0.0	13.00	22.50
Code	2	0.2	5	0.0	0.00	11.20
Social Networking Site	1	0.1	1	0.0	0.00	15.00
Governmental body	1	0.1	3	0.0	73.00	55.00
Library	1	0.1	1	0.0	38.00	37.00
TOTAL	882		15,585			

A massive concentration of URL citations from academic websites was detected, in particular, those of publishers (21.8% of all source domain names, generating 64.2% of all URL citations) and higher education institutions (8.4% of domain names, generating 14.6% of all URL citations). While the presence of organizations and companies is also strong (10 and 9.3% of domain names, respectively), the number of URL citations provided by these sources was only moderate (2.7 and 3.6%, respectively). The number of URL citations from encyclopaedias is also notable (4.9% of all URL citations received).

**Fig. 7:**
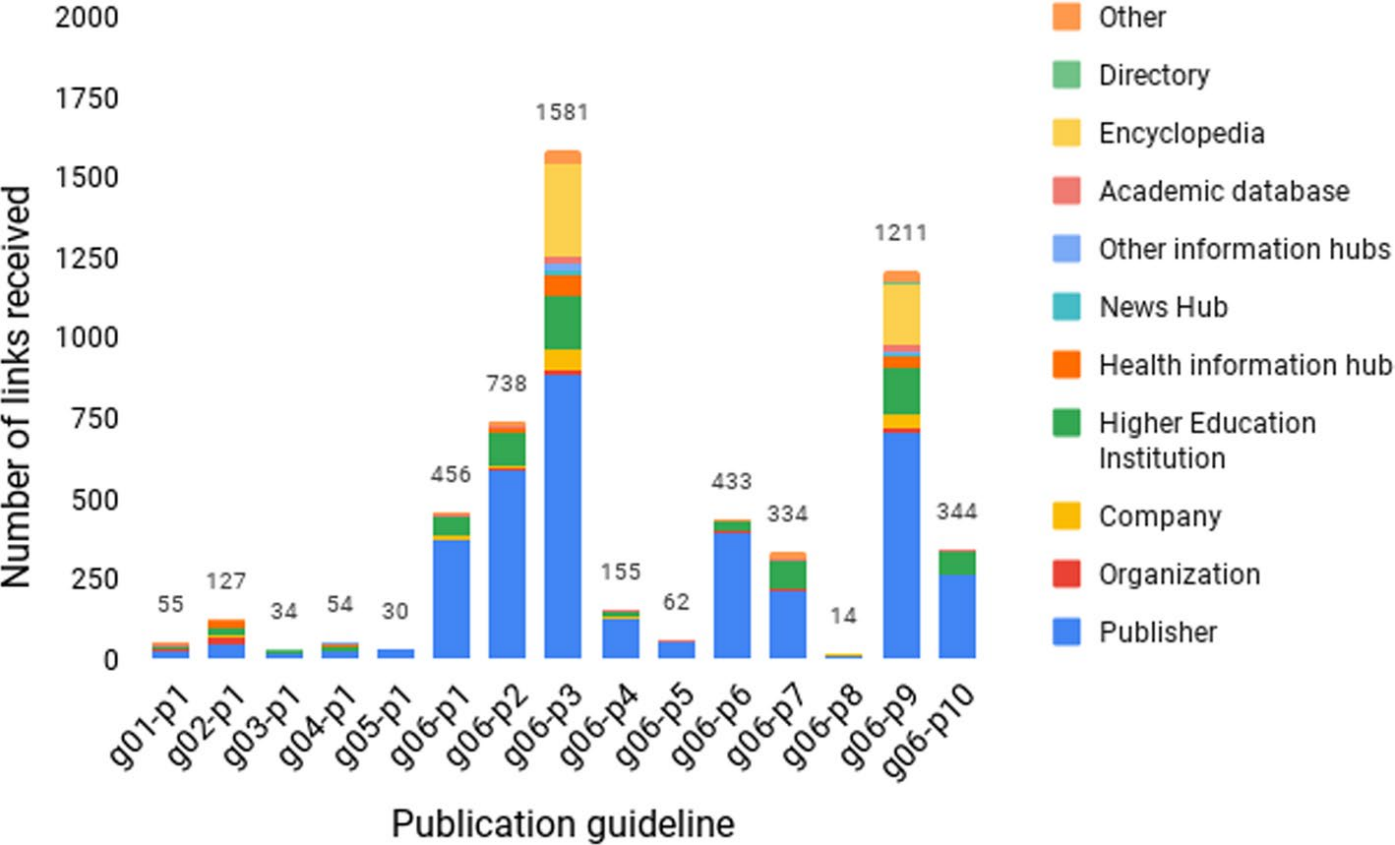
Number of URL citations received according to the entity type for articles related to the CONSORT 2010 statement

While the publishers’ websites were the origin of most URL citations, the distribution of links by entity type differed from article to article. For example, in the case of CONSORT 2010, a remarkable number of URL citations received by G06-P3 and G06-P9 originated from encyclopaedias, a phenomenon not observed in the remaining publications describing the same reporting guidelines (Fig. 7). The distribution of URL citations by entity category for all publications can be consulted in the supplementary material (Appendix E).

BioMed Central and Springer (both part of the Springer Nature Group) are the source domain names that provided the highest number of URL citations to the set of articles considered (39.9 and 13.3% of all URL citations, respectively) (Table 5). These two publishers also linked to the highest number of different articles (e.g., BioMed Central provided links to 58 of the 64 articles analysed). Other major domain names in the Springer Nature Group include Nature (150 URL citations), BMC Medicine (88 URL citations),^15^ and SpringerOpen (75 URL citations). However, data offered in Table 5 show domain names. Consequently, they are not related to specific journals necessarily. For example, nature.com includes publications from different journals, all hosted under the same domain name.

**Table 5:** Top source domain names

Domain names	Number of URL citations	%	Number of articles	TF	CF	Entity type
biomedcentral.com	5279	33.9	58	72	65	Publisher
springer.com	2075	13.3	48	75	73	Publisher
beds.ac.uk	1583	10.2	39	56	48	Higher Education Institution
hindawi.com	465	3.0	35	59	57	Publisher
wikipedia.org	318	2.0	13	93	97	Encyclopaedia
lshtm.ac.uk	304	2.0	11	65	54	Higher Education Institution
frontiersin.org	257	1.6	41	43	58	Publisher
equator-network.org	208	1.3	22	40	46	Organization
bitbybitbook.com	208	1.3	1	28	29	Publisher
jamanetwork.com	190	1.2	30	43	56	Publisher
bmj.com	160	1.0	20	83	66	Publisher
nature.com	150	1.0	37	82	68	Publisher
lww.com	126	0.8	26	63	63	Publisher
lablynx.com	124	0.8	10	19	32	Company
plos.org	101	0.6	44	81	69	Publisher
biomedcentral.eu	88	0.6	27	0	10	Publisher
hmoob.press	79	0.5	12	0	4	Encyclopaedia
springeropen.com	75	0.5	21	39	52	Publisher
nih.gov	75	0.5	43	95	89	Health Government body
mdwiki.org	63	0.4	6	22	26	Health information hub
researchprotocols.org	62	0.4	5	36	36	Publisher
theadx.net	60	0.4	1	0	16	Company
mdpi.com	54	0.3	22	59	60	Publisher
aerzteblatt.de	47	0.3	5	59	63	Publisher
linksmedicus.com	45	0.3	5	16	39	Academic database

Wikipedia also has a notable presence, generating 318 links to 13 different articles, as does the EQUATOR network (208 links); yet, the number of articles linked from this source (22) was lower than expected. Other publishers (e.g., Hindawi, Frontiers, JAMA Network, BMJ, and PLoS) were among the top source domain names generating URL citations to reporting guideline articles (Table 5).

The analysis identified a number of other special cases. For example, the University of Bedfordshire (beds.ac.uk) generated 10.2% of all URL citations; however, a fine-grained analysis revealed that the links were generated by journal mirrors,^16^ specifically a mirror of BMC Medicine hosted on the University of Bedfordshire website.^17^

Other domain names were found to link massively to the same guideline article– for example Bitbybitbook.com (a book openly accessible), which generated 208 URL citations to CONSORT-2010 article G6-P06 (Schulz et al., 2010b).

At the object level, 18 object genres and six potential purpose categories were identified (Table 6). A brief description of the scope of each genre can be consulted in the supplementary material (Appendix F). Object genres were detected for 9527 online objects of the 15,585 obtained following the debugging process (for further details, see Fig. 9).

**Table 6:** Number of URL citations according to the source object genre

Source object genre	Potential purpose	Number of URL citations	%
Publication/Article	Scientific	7023	73.72
Publication/Article/Mirror	Scientific	1126	11.82
Bibliographic record	Functional	327	3.43
Curated reference list	Promotional	279	2.93
Wiki entry	Informational	192	2.02
Publication/Book	Scientific	163	1.71
News	Informational	144	1.51
Post/Blog	Informational	78	0.82
Author guidelines	Informational	68	0.71
Personal webpages	Promotional	61	0.64
Resource list	Informational	30	0.31
Data report/Altmetric	Functional	17	0.18
Post/Forum	Discussion	6	0.06
Institutional information	Informational	3	0.03
Data report/SEO tool	Functional	3	0.03
Teaching material	Alternative impact	3	0.03
Publication/Article/Summary	Scientific	2	0.02
Tweet	Informational	2	0.02
Total		9527	100

URL citations were primarily created for scientific-related reasons (87.27% of all links). Most of these links were citations from other scientific publications, either from the publishers’ websites or from journal mirrors. The remaining source object genres were residual, with a notable presence of informational (e.g., links in news, posts and encyclopaedias) and promotional (e.g., links in curated references lists and personal websites) links.

Finally, the presence of links in bibliographic records (e.g., repositories) or data reports (e.g., Altmetrics data providers or search engine optimization – SEO – tools) was also noted. These links are created automatically as they respond to other functional tasks.

### Reputation of websites linking to reporting guidelines

**Fig. 8:**
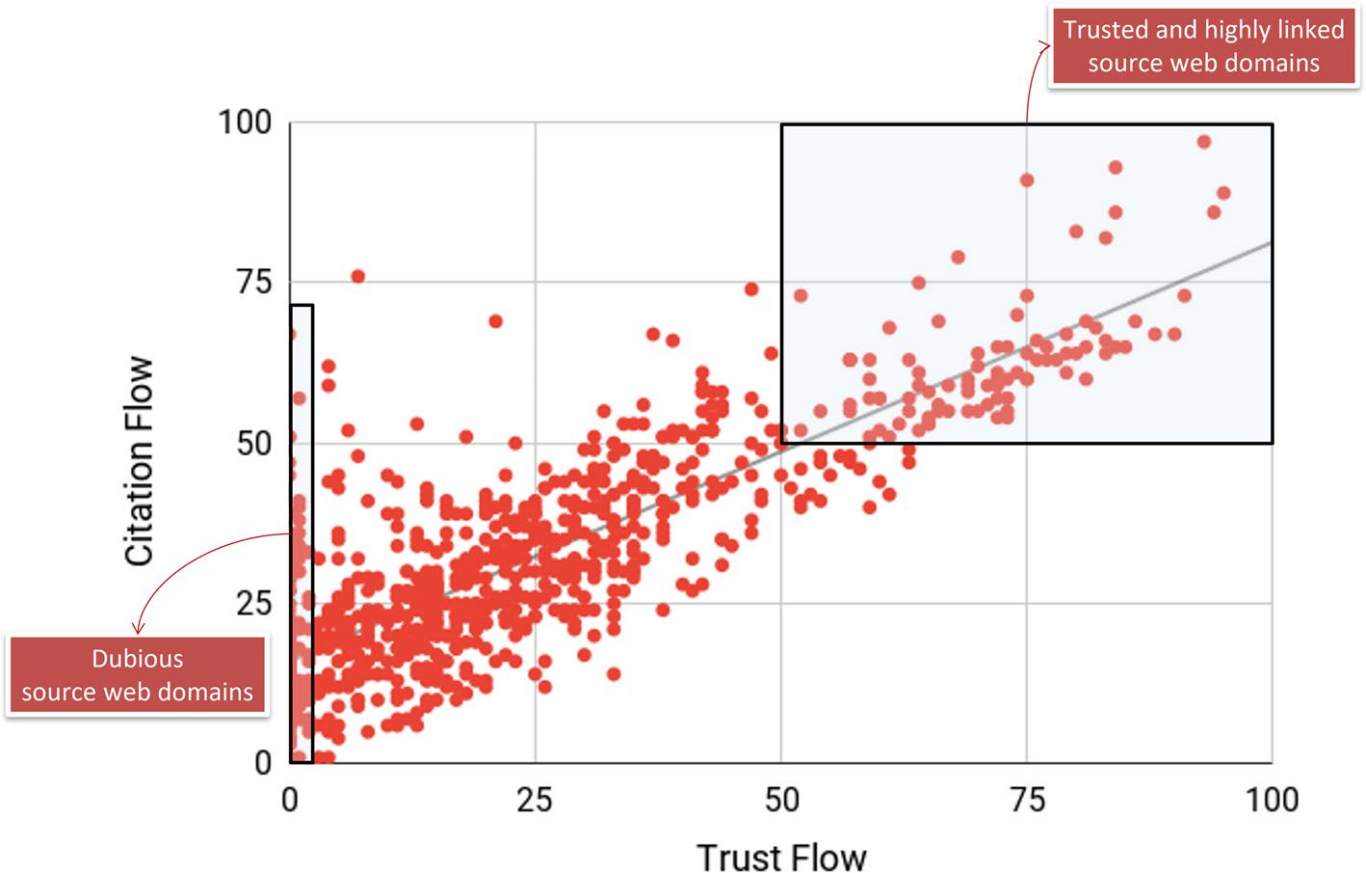
Scatter plot of Trust Flow and Citation Flow values for source domain names

The breadth of reputation of the 64 articles related to the CONSORT and SPIRIT statements reached a figure of 91 domain names, generating 9637 URL citations (that is, 61.8% of all links received by these articles). These domain names constitute the most reputed domains from which the reporting guidelines received URL citations (Fig. 8). Publishers and higher education institutions recorded high Trust Flow values (see Tables 4 and 5 for values at the entity type and domain name, respectively). These values can be considered significant given the high number of source domain names tagged in each of these two categories.

The Trust Flow and Citation Flow values recorded were strongly correlated (Spearman *R* = 0.77; *p*-value: < 0.0001) (i.e., they present a balanced Flow Metrics profile). However, the values of these two flow metrics were unbalanced in several domain names. More specifically, the Citation Flow value of 124 domain names was twenty times that of their Trust Flow value, suggesting that the real reputation of these source domain names is dubious.

### Decay of URL citations to reporting guidelines

The application of the DVAF method dramatically reduced both the number of URL citations to articles (96%; from 240,128 links to 9527) and the number of linking domain names (95%; from 8064 to 421).

**Fig. 9:**
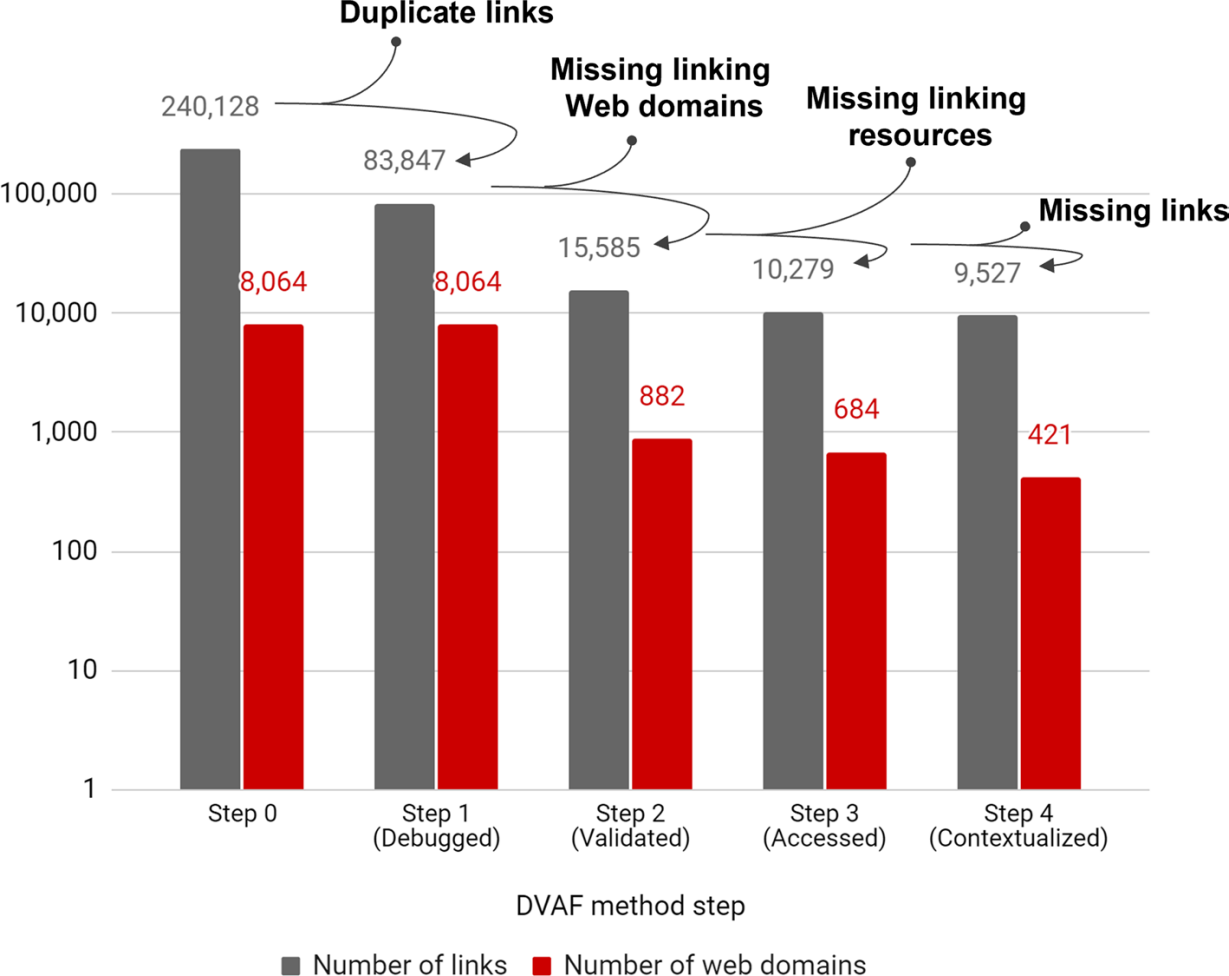
Reduction in number of URL citations with each successive step of the DVAF method

While the first step (Debug) mainly eliminated duplicate links, the remaining steps highlighted the large-scale obsolescence of link data. The second step (Validate) primarily detected domain names that had once existed, but which are no longer active (6987 domain names providing 83,847 URL citations). The third step (Access) largely identified source objects that had existed at some time, but which had subsequently disappeared. More specifically, 192 objects (providing 308 URL citations) returned a 404 HTML code (Not Found), while 902 objects (providing 1307 URL citations) could not be accessed. Finally, the fourth step (Find) chiefly detected links that had once existed, but which had since disappeared. Thus, no URL citations were found in 415 objects. This gradual process of reduction with the application of successive steps is illustrated in Fig. 9.

The reduction in the number of URLs is not limited to poor quality or non-reputable domain names. The DVAF method, for example, reduced the number of URL citations from BioMed Central by 26.8% (from 5279 to 3860) and those from Springer by 27.9%.

The URL decay effect presented by the top ten linking domain names is shown in Table 7, where we can observe that for these domain names the outcomes remained largely unchanged from steps 3 to 4. In addition, a remarkable presence of publishers is found (BioMed Central, Springer, Hindawi, Frontiers in, and JAMA Network). The disappearance of informative webpages or huge changes on the domain name structure might explain the URL decay on these websites. The loss of links on Wikipedia is attributed to the dynamic and social nature of the site.

**Table 7:** Decay URL citations for top linking domain names

Domain name	Number of URL citations		
	Step 2	Step 3	Step 4
biomedcentral.com	5,279	3,860	3,859
springer.com	2,075	1,496	1,496
beds.ac.uk	1,583	1,119	1,119
hindawi.com	465	364	364
wikipedia.org	318	82	80
lshtm.ac.uk	304	208	190
frontiersin.org	257	164	164
equator-network.org	208	43	43
bitbybitbook.com	208	161	161
jamanetwork.com	190	151	151

## Discussion

In this study, we have presented a cross-sectional analysis of web-based data for articles addressing the CONSORT and SPIRIT statements. The online impact of these articles has been determined using link analysis, for which a tailored cleansing data process (DVAF method) has been designed, developed, and applied. Thanks to the application of this method, it has been possible to identify different facets of the online impact of these reporting guidelines for randomised trials and associated protocols.

### About the online impact of reporting guidelines

We found that reporting guidelines in this study received 15,582 URL citations (RQ1a). The CONSORT 2010 for parallel-group randomised controlled trials described in 10 articles were the most cited reporting guidelines (with 5328 URL citations or links) from other online objects, particularly in comparison with other guidelines or extensions included in the analyses. In our opinion, these results can be explained by the fact that a large number of CONSORT/SPIRIT extensions or adaptations seem less well known among authors, reviewers and editors, since many of them are not being systematically incorporated into the journals’ instructions to authors. For example, a previous study (Shamseer et al., 2016) examined the online “Instructions to Authors” of 168 high Impact Factor medical journals between July and December 2014. Sixty-three percent (106/168) of the included journals mentioned CONSORT in their “Instructions to Authors" and only 22 of the journals (13%) mentioned any of the CONSORT extensions published at the time of searching.

The results evidence a scattered web impact, reflected by the existence of different URL IDs referring to the same article (RQ1b). While use of the DOI URL ID is generalized, 17% of links (2660) would have been missed if DOI were the sole URL ID employed. In fact, for a few recent articles (those published in 2020 and 2021), the journal URL ID was the one with the most links. A plausible explanation for this is that these articles would not have had sufficient time to obtain bibliographic citations, a significant source of DOI links. Moreover, the scattered effect of the online impact is accentuated in the case of those guidelines described in more than one article, where the existence of different distributions of links according to each URL ID is apparent. Consequently, the article-level link analysis should not ignore URL IDs, other than that corresponding to the DOI.

The online impact of the articles (considering all links received regardless of the URL ID linked) shows a strong and significantly positive correlation both with the number of citations received (scientific impact) and the set of Altmetrics analysed (wider impact), especially the number of Mendeley readers (RQ2). Consequently, counting the total number of links received per article (after cleansing the data in accordance with the DVAF method) might be informative of the scientific impact of articles, and indirectly, of the guidelines described. For recent articles, links can also be used as potential early predictors of their future scientific impact.

An absence of any correlation with Facebook and Twitter counts has been detected. A plausible explanation for this performance would appear to be related to the year of publication, as most of the articles were published long before the launch of these networking sites, and even before PlumX started collecting data. More recent articles (e.g., CONSORT extension for reporting randomised trials of social and psychological interventions [CONSORT-SPI]: G23 in 2018, and CONSORT extension for reporting Adaptive designs [ACE]: G30 in 2020) record considerably higher Facebook and Twitter counts, reinforcing this hypothesis.

The average number of single domain names linking to guideline articles is low (34.6), with 50% of all articles receiving links from fewer than 29 different domain names. This result points to the concentration of source domain names linking to articles describing reporting guidelines (RQ3).

While the diversity of linking entities is quite massive (38 different entity types were found, both academic and non-academic), the online impact is heavily concentrated (RQ4). In general, a strong clustering of URL citations was detected from academic websites, particularly from publishers (64% of all URL citations, and 22% of all source domain names), and higher academic institutions (15% of all URL citations, and 8% of domain names), in contrast to other institutions (e.g., government bodies and research institutes represented less than 2% of URL citations, and 1% of domain names). We interpret the different URL patterns between websites might potentially reflect editorial policies and practices, and those institutional websites incorporating reporting guidelines could be considered enablers high-quality reporting standards for randomised trial reports and protocols. Perhaps the most striking results the low representation of research funders, for their important role in the promotion and development of clinical research, with the sole exception of the U.S. National Institutes of Health (NIH), placed on the list of top source domain names.

Most URL citations (85.4%) come from articles, with a scientific purpose (citation) accounting for the creation of the link. This result might explain the high online impact of the DOI URL ID as well as the strong correlation between URL citations and citations (i.e., the DOI is incorporated as part of the reference that cites the article describing the reporting guideline). The existence of URL citations from news sources, encyclopaedias, research centres, personal websites and health information portals testifies to their wider impact, driven in this instance by motives of an informational and promotional nature.

These results are obviously limited by the categorization process carried out. Despite the large number of entity types detected (38), most websites belong to publishers (publishing groups and academic journals) that are easily identifiable. However, the presence of publishers is probably underrepresented, since all the journals hosted by universities have been counted as higher education institutions instead of publishers, since the general domain name belongs to the whole institution, being this issue the main limitation of the entity categorization performed. The subsequent classification of source object genres regardless the entity type minimizes this effect, adding accurateness to the link analysis carried out.

The reputation of the linking websites is highly suspecting (RQ5). If we consider the original 240,128 URL citations before debugging, 84.2% of these links come from dubious websites and fake domain names with a Trust Flow value of 0. After debugging, only 10.3% of all source domain names (*n *= 91) achieve Trust Flow and Citation Flow values of at least 50. These reputed domain names generate a significant percentage of all the URL citations (61.8%) received by the reporting guidelines. This means that the actual incidence of low-quality source domain names is low. A few source domains, however, exhibit an unbalanced Flow Metric profile, which might be because they were built for promotional or SEO purposes, as linking academic publications enhances their credibility in the eyes of readers and search engines. This issue clearly suggests that dubious websites use research objects as part of their commercial link strategies, an issue already identified in previous studies (Orduña-Malea, 2021).

The results also highlight a large-scale URL decay (RQ6). If we consider steps 2–4 of the DVAF method (those involved in the URL decay), 88.6% of linking domain names and 94.8% of URL citations could not be accessed at the time of the analysis. Non-accessible domain names were mainly associated with fake websites created for the purposes of SEO and whose lifespan is ephemeral, while most non-accessible objects were associated with changes made to reputable websites. Finally, the presence of URL citations in webpages that could not be found can be attributed to the appearance of links in ephemeral locations (e.g., sidebars, comments, and blog feeds).

### About the dissemination of reporting guidelines

This study provides considerable information that can be used to very diverse purposes, such as activities related to promote health research conduct, reporting, and scientific writing and peer-review. For example, journals and publishers have an important role to play in the dissemination and implementation of the reporting guidelines. The inclusion of hyperlinks and full citations to articles (e.g., DOI and journal URL IDs) in the journals’ publication instructions is a recommended course of action in this regard. Similarly, those responsible for writing and promoting the guidelines need to explore and improve different implementation strategies aimed at increasing the adoption of the recommendations by authors, reviewers, and journal editors. In addition, enlisting the support of other relevant actors, including professional societies and organizations and funding agencies, would also appear to be crucial, while the publication of supporting articles (e.g., documents, letters, comments, editorials, and translations of “Explanation and Elaboration” papers) and the use of web-based dissemination channels (journal websites, guideline groups, and international initiatives such as EQUATOR) would do much to help in their dissemination.

While the results reported in this study are limited to the CONSORT and SPIRIT initiatives, the analytical methods described here can be applied to the measurement of other leading reporting guidelines in health research, including, for example, the PRISMA (Preferred Reporting Items for Systematic Reviews and Meta-Analyses) statement (Moher et al., 2009; Page et al., 2021). The enhanced dissemination and implementation of reporting guidelines would undoubtedly serve to improve the quality and transparency of articles reporting randomised trials, which, in turn, would have an impact on future research.

### About the DVAF method

The DVAF (Debug, Validate, Access, and Find) method described here has been used to analyse the online impact of articles describing trial reporting guidelines. However, it should be borne in mind that this method relies on the measurement of links, so that all guidelines mentioned by other procedures (e.g., title textual mention) are not captured (Thelwall, 2011).

The method does, nevertheless, facilitate the cleaning of link data. The ‘Debug’ step eliminated duplicate links while the ‘Validate’ step filtered out all dubious websites, and given the huge percentage of links cleaned, these steps must be considered essential for academic link analysis. Once the link data had been debugged, the results captured primarily scientific impact (i.e., links from articles) and, to a lesser extent, wider academic impact (i.e., informational and promotional links). The URL citations received by the articles exhibited a strong correlation both with the number of citations received and a set of alternative metrics, thus reinforcing their value as supplementary sources of impact, especially for recent articles (with URLs being created faster than citations).

On the downside, the DVAF method is time-consuming (above all steps 2 and 4) and while some steps could be automated, the validation and access stages require human intervention. Here, a set of just 64 articles has been analysed, but the analysis of large sets of documents can be considerably more complex. In particular, the categorization of entities is difficult due to the existence of complex websites and this step is not, therefore, readily automated. Additionally, the presence of fraudulent websites introduces a series of inherent errors in the link analysis process, and while the proposed method considerably reduces their number, it does not eliminate them entirely. In this regard, the Trust Flow has shown itself to be a useful metric for filtering out dubious websites and enhancing academic websites, as reported previously in the literature (Orduña-Malea, 2021).

The obsolescence of link data (URL decay) constitutes an additional limitation, as the ‘Access’ and ‘Find’ steps have both highlighted. Entire websites, particular online objects or even links can disappear over time. The DVAF method allows missing links to be identified, but the researcher must decide whether to consider only currently active links or all the links that existed at one time or another. The results reported here indicate that even links from academic websites can disappear, evidencing results from classic studies (e.g., Klein et al., 2014; Koehler, 1999; Payne & Thelwall, 2007). This problem is a major limitation of link-based impact analysis, which is partially solved by using the historic index offered by Majestic.

Another limitation of the method described here is its dependence on one specific link data source, in this case Majestic. Each data source has its strengths and weaknesses, but ultimately each defines its own specific method of data collection (as is equally true of bibliographic databases). Although this issue can be addressed in part by designing one’s own crawler, this solution is not readily implemented, as Majestic's worldwide coverage (and system maintenance) is technically and economically difficult to achieve. Considering these potential limitations, the DVAF method has been designed and applied to measure the online impact of the CONSORT and SPIRIT reporting guidelines, breaking down the impact into six facets, being the results obtained highly satisfactory. In addition, due to the systemic characteristics of the DVAF method, it can be extrapolated to be used in any link analysis, regardless the nature of the source and target objects.

## Conclusions

Our analysis represents the first attempt to systematically evaluate the impact of reporting guidelines for randomised trial reports and protocols using methods and tools from link analysis. In light of our results, it is concluded that the online impact of CONSORT and SPIRIT could be improved. The study has served to identify reporting guidelines for randomised trial reports and protocols, key actors disseminating them (domain names, websites, source objects), and impact (citations, URL citations, Altmetrics).

In our opinion, these findings could be used to strengthen reporting guidelines uptake to increase value and reduce waste from incomplete or unusable randomised trial reports and protocols.

Finally, a new link analysis method (DVAF) has been designed and tested, aimed at cleaning link data. The method has been shown to be efficient to decompose the online impact into different facets (scattering, degree of similarity, broadness, diversity, and URL decay), increasing the accuracy of link analysis.

## Supplementary Information

Below is the link to the electronic supplementary material.

Supplementary file1 (DOCX 7802 KB)

## Footnotes

1 https://dev.w3.org/html5/spec-LC/.

2 Each online object is referred to by means of its URL.

3 For example, the URLs “https://www.bmj.com/content/361/bmj.k1079” and “https://doi.org/10.1136/bmj.k1079” point to the same research object (a journal article), and each of them receive links from different sources.

4 http://www.consort-statement.org.

5 https://www.spirit-statement.org.

6 https://www.equator-network.org.

7 https://majestic.com.

8 https://majestic.com/help/glossary.

9 Status codes are issued by a server in response to a client request made to the server.

10 https://gephi.org.

11 https://plumanalytics.com.

12 Clinical guidelines to the CONSORT 2010 statement published in BMJ (Schulz, 2010a): https://plu.mx/a/1jr0-Zals0nbC0qAsG9mV4myUpvyCq-xkm59jlAe1to/clinical_citation.

13 Policy guidelines to the CONSORT 2010 statement published in BMJ (Schulz, 2010a): https://plu.mx/a/1jr0-Zals0nbC0qAsG9mV4myUpvyCq-xkm59jlAe1to/policy_citation.

14 https://dev.elsevier.com/documentation/PlumXMetricsAPI.wadl.

15 biomedcentral.eu is a mirror of https://bmcmedicine.biomedcentral.com.

16 A mirror website is a replica of another website with a different URL to that of the original site, but which hosts identical or near-identical content.

17 https://0-bmcmedicine-biomedcentral-com.brum.beds.ac.uk/articles/10.1186/1741-7015-12-69.
