# Supplementary material for: Evaluating the online impact of reporting guidelines for randomised trial reports and protocols: a cross-sectional web-based data analysis of CONSORT and SPIRIT initiatives
Source: Scientometrics. 2022 Oct 17;128(1):407–40. doi: 10.1007/s11192-022-04542-z (PMC9574182; doi:10.1007/s11192-022-04542-z)

**Supplementary material**

**Evaluating the online impact of reporting guidelines for randomised trial reports and protocols: a cross-sectional web-based data analysis of CONSORT and SPIRIT initiatives**

**Enrique Orduña-Malea^1^** [
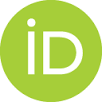
](https://orcid.org/0000-0002-1989-8477)**, Adolfo Alonso-Arroyo^2,3^** [
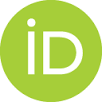
](https://orcid.org/0000-0002-1989-8477)**, José-Antonio Ontalba-Ruipérez^4^** [
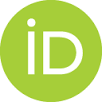
](http://orcid.org/0000-0002-7465-6462)**, and Ferrán Catalá-López^5,6,7^** [
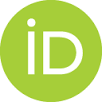
](https://orcid.org/0000-0002-3833-9312)

^1^Department of Audiovisual Communication, Documentation and History of Art, Universitat Politècnica de València, Valencia, Spain

✉ enorma@upv.es

^2^Department of History of Science and Documentation, University of Valencia, Valencia, Spain

^3^UISYS, Joint Research Unit CSIC–University of Valencia, Valencia, Spain

✉ Adolfo.Alonso@uv.es

^4^Department of Audiovisual Communication, Documentation and History of Art, Universitat Politècnica de València, Valencia, Spain

✉ joonrui@upv.es

^5^Department of Health Planning and Economics, National School of Public Health, Institute of Health Carlos III, Madrid, Spain

^6^Department of Medicine, University of Valencia/INCLIVA Health Research Institute and CIBERSAM, Valencia, Spain

^7^Knowledge Synthesis Group, Clinical Epidemiology Program, Ottawa Hospital Research Institute, Ottawa, Canada

✉ ferran_catala@outlook.com

**Appendix A** 2

Dataset of guidelines and article guidelines

**Appendix B** 3

Number of URL citations received by articles according to each URL ID

**Appendix C** 5

Altmetric data (PlumX)

**Appendix D** 7

Source entity types

**Appendix E** 9

Number of linking domain names per entity type for each article

**Appendix F** 11

Object genres

**Appendix A**

Dataset of guidelines and article guidelines

|  | Reporting guideline | Number  of articles |
| --- | --- | --- |
| G1 | CONSORT 2010 | 11 |
| G2 | STRICTA | 4 |
| G3 | CONSORT-AI | 3 |
| G4 | CONSORT 2001 | 3 |
| G5 | SPIRIT-AI | 3 |
| G6 | CONSORT-Pilot and Feasibility | 2 |
| G7 | SPIRIT 2013 | 2 |
| G8 | CONSORT-SPI | 2 |
| G9 | CONSORT-Abstracts | 2 |
| G10 | CONSORT-Non-pharmacologic treatment 2008 | 2 |
| G11 | ACE | 2 |
| G12 | CONSORT-CENT | 2 |
| G13 | CONSORT-Herbal | 2 |
| G14 | Simulation Research | 1 |
| G15 | TIDIeR-PHP | 1 |
| G16 | SPIRIT-PRO | 1 |
| G17 | CONSORT-Cluster | 1 |
| G18 | CONSORT 1996 | 1 |
| G19 | CONSORT-EHEALTH | 1 |
| G20 | CENT for TCM | 1 |
| G21 | CONSORT-Equity | 1 |
| G22 | CONSERVE | 1 |
| G23 | CONSORT-Harms | 1 |
| G24 | CONSORT-CHM 2017 | 1 |
| G25 | CONSORT crossover | 1 |
| G26 | CONSORT-Within person | 1 |
| G27 | CONSORT-Non-inferiority | 1 |
| G28 | SPENT | 1 |
| G29 | CONSORT multi-arm | 1 |
| G30 | CONSORT-C | 1 |
| G31 | CONSORT-Non-pharmacologic treatment 2017 | 1 |
| G32 | SPIRIT-TCM | 1 |
| G33 | CONSORT SW-CRT | 1 |
| G34 | TIDIeR | 1 |
| G35 | CONSORT-Pragmatic Trials | 1 |
| G36 | TIDieR-Placebo | 1 |
| G37 | CONSORT-PRO | 1 |
| G38 | CONSORT-ROUTINE | 1 |
|  | TOTAL | 65 |

**Appendix B**

Number of URL citations received by articles according to each URL ID

| Publication | Journal | URL IDs | | | | | Years |
| --- | --- | --- | --- | --- | --- | --- | --- |
|  |  | **DOI** | **Journal** | **PMC** | **PubMed** | **ALL** |  |
| G01-p1 | British medical journal | 36 | 17 |  | 2 | 55 | 3 |
| G02-p1 | Jama | 61 | 64 |  | 2 | 127 | 3 |
| G03-p1 | British medical journal | 19 | 15 |  |  | 34 | 4 |
| G04-p1 | British medical journal | 45 | 5 | 3 | 1 | 54 | 4 |
| G05-p1 | Annals of internal medicine | 29 |  |  | 1 | 30 | 4 |
| G06-p1 | Annals of internal medicine | 441 | 12 |  | 3 | 456 | 11 |
| G06-p2 | BMC medicine | 682 | 48 | 3 | 5 | 738 | 11 |
| G06-p3 | British medical journal | 1,218 | 50 | 200 | 113 | 1,581 | 11 |
| G06-p4 | Journal of clinical epidemiology | 154 | 1 |  |  | 155 | 11 |
| G06-p5 | Obstetrics and gynecology | 60 | 1 |  | 1 | 62 | 11 |
| G06-p6 | PLoS Medicine | 424 | 6 | 3 |  | 433 | 11 |
| G06-p7 | Trials | 272 | 57 | 4 | 1 | 334 | 11 |
| G06-p8 | Lancet | 14 |  |  |  | 14 | 11 |
| G06-p9 | British medical journal | 970 | 38 | 134 | 69 | 1,211 | 11 |
| G06-p10 | Journal of clinical epidemiology | 339 |  |  | 5 | 344 | 11 |
| G07-p1 | Annals of internal medicine | 1,181 | 19 | 5 | 9 | 1,214 | 8 |
| G07-p2 | British medical journal | 924 | 24 | 18 | 10 | 976 | 8 |
| G08-p1 | Pilot and feasability studies | 187 | 37 |  | 7 | 231 | 5 |
| G08-p2 | British medical journal | 206 | 40 |  | 3 | 249 | 5 |
| G09-p1 | Journal of the society for simulation in healthcare | 10 | 1 |  | 2 | 13 | 5 |
| G10-p1 | British medical journal | 16 | 8 |  |  | 24 | 6 |
| G10-p2 | British medical journal | 13 |  |  |  | 13 | 6 |
| G11-p1 | British medical journal | 862 | 64 |  | 9 | 935 | 7 |
| G12-p1 | Annals of internal medicine | 259 | 16 |  | 44 | 319 | 17 |
| G13-p1 | Journal of medical internet research | 143 | 51 | 5 | 2 | 201 | 10 |
| G14-p1 | Jama | 130 | 12 |  | 1 | 143 | 8 |
| G15-p1 | Lancet | 3 |  |  |  | 3 | 11 |
| G16-p1 | British medical journal | 467 | 13 | 52 | 27 | 559 | 13 |
| G17-p1 | Lancet | 87 | 5 |  | 3 | 95 | 13 |
| G17-p2 | PLoS Medicine | 184 | 8 | 45 | 29 | 266 | 13 |
| G18-p1 | Annals of internal medicine | 87 | 3 |  | 2 | 92 | 4 |
| G19-p1 | Annals of internal medicine | 98 | 6 |  | 5 | 109 | 15 |
| G19-p2 | Journal of clinical epidemiology | 27 |  |  | 2 | 29 | 15 |
| G20-p1 | British medical journal | 474 | 6 |  | 23 | 503 | 9 |
| G21-p1 | Jama | 299 | 5 |  | 1 | 305 | 9 |
| G22-p1 | PLoS Medicine | 183 | 2 | 8 | 2 | 195 | 11 |
| G22-p2 | Journal of alternative and complementary medicine | 23 |  |  |  | 23 | 11 |
| G22-p3 | Journal of evidence-based medicine | 129 |  |  | 1 | 130 | 11 |
| G22-p4 | Acupuncture in medicine | 65 |  | 1 | 1 | 67 | 11 |
| G23-p1 | Trials | 31 | 34 | 1 | 2 | 68 | 3 |
| G23-p2 | Trials | 35 | 23 | 2 | 5 | 65 | 3 |
| G24-p1 | Chinese journal of integrative medicine | 15 |  |  |  | 15 | 2 |
| G25-p1 | British medical journal | 7 |  |  | 3 | 10 | 1 |
| G26-p1 | Complementary therapies in medicine | 4 |  |  | 1 | 5 | 2 |
| G27-p1 | British medical journal | 33 | 35 | 1 | 1 | 70 | 2 |
| G28-p1 | Jama | 18 | 35 |  |  | 53 | 2 |
| G29-p1 | British medical journal | 85 | 60 | 3 | 1 | 149 | 3 |
| G30-p1 | British medical journal | 29 | 68 | 5 | 28 | 130 | 1 |
| G30-p2 | Trials | 21 | 5 | 4 | 26 | 56 | 1 |
| G31-p1 | British medical journal | 21 | 50 | 1 | 8 | 80 | 1 |
| G31-p2 | Nature medicine | 28 | 42 |  | 9 | 79 | 1 |
| G31-p3 | Lancet digital health | 14 | 85 |  | 5 | 104 | 1 |
| G32-p1 | Nature medicine | 28 | 246 |  | 31 | 305 | 1 |
| G32-p2 | British medical journal | 18 | 50 |  | 29 | 97 | 1 |
| G32-p3 | Lancet digital health | 14 | 45 |  | 26 | 85 | 1 |
| G33-p1 | Jama | 89 |  |  | 19 | 108 | 25 |
| G34-p1 | Annals of internal medicine | 134 | 1 |  | 2 | 137 | 20 |
| G34-p2 | Jama | 272 | 3 |  |  | 275 | 20 |
| G34-p3 | Lancet | 554 |  |  | 5 | 559 | 20 |
| G35-p1 | Annals of internal medicine | 56 |  |  | 19 | 75 | 13 |
| G35-p2 | Annals of internal medicine | 568 | 9 |  | 21 | 598 | 13 |
| G36-p1 | PLoS Medicine | 21 | 14 | 1 | 2 | 38 | 1 |
| G37-p1 | British medical journal | 8 | 14 | 9 | 27 | 58 | 0 |
| G38-p1 | Jama | 1 | 41 |  | 2 | 44 | 0 |

Years: number of years since the publication of the article. The value 0 corresponds to 2021.

G6-P11 is excluded because this article doesn’t have a URL-based DOI.

**Appendix C**

Altmetric data (PlumX)

| PUBLICATION | ABSTRACT  VIEWS | CITATIONS | CLINICAL  CITATIONS | EXPORTS  SAVES | FACEBOOK  COUNT | POLICY  CITATIONS | READER  COUNT | TWEET  COUNT | URL CITATIONS |
| --- | --- | --- | --- | --- | --- | --- | --- | --- | --- |
| G1-P01 | 0 | 55 | 0 | 0 | 8 | 2 | 105 | 127 | 55 |
| G2-P01 | 301 | 242 | 1 | 54 | 55 | 1 | 325 | 120 | 127 |
| G3-P01 | 0 | 47 | 0 | 0 | 0 | 3 | 93 | 193 | 34 |
| G4-P01 | 0 | 90 | 0 | 0 | 129 | 1 | 157 | 101 | 54 |
| G5-P01 | 8 | 90 | 2 | 4 | 2 | 1 | 52 | 4 | 30 |
| G6-P01 | 2,152 | 2,033 | 16 | 163 | 0 | 64 | 615 | 0 | 456 |
| G6-P02 | 216 | 1,942 | 2 | 37 | 3 | 25 | 529 | 6 | 738 |
| G6-P03 | 326 | 3,729 | 15 | 37 | 10 | 97 | 2,583 | 7 | 1,581 |
| G6-P04 | 377 | 551 | 1 | 21 | 0 | 19 | 292 | 0 | 155 |
| G6-P05 | 1 | 210 | 1 | 0 | 0 | 2 | 300 | 0 | 62 |
| G6-P06 | 272 | 785 | 3 | 34 | 0 | 37 | 539 | 2 | 433 |
| G6-P07 | 127 | 568 | 0 | 15 | 48 | 8 | 239 | 1 | 334 |
| G6-P08 | 1 | 81 | 0 | 0 | 0 | 3 | 104 | 2 | 14 |
| G6-P09 | 126 | 2,974 | 14 | 17 | 64 | 60 | 2,852 | 31 | 1,211 |
| G6-P10 | 1,275 | 1,202 | 3 | 32 | 1 | 14 | 815 | 2 | 344 |
| G7-P01 | 1,846 | 2,261 | 3 | 58 | 715 | 62 | 1,228 | 7 | 1,214 |
| G7-P02 | 110 | 1,628 | 5 | 15 | 29 | 36 | 1,577 | 93 | 976 |
| G8-P01 | 24 | 366 | 0 | 7 | 65 | 1 | 552 | 58 | 231 |
| G8-P02 | 0 | 587 | 1 | 0 | 247 | 2 | 620 | 107 | 249 |
| G9-P01 | 0 | 115 | 0 | 2 | 0 | 1 | 69 | 6 | 13 |
| G10-P01 | 21 | 95 | 2 | 1 | 0 | 4 | 124 | 37 | 24 |
| G10-P02 | 16 | NA | 1 | 1 | 123 | 2 | 105 | 32 | 13 |
| G11-P01 | 227 | 2,964 | 9 | 29 | 136 | 92 | 2,567 | 311 | 935 |
| G12-P01 | 767 | 1,005 | 27 | 48 | 0 | 61 | 571 | 4 | 319 |
| G13-P01 | 1,015 | 853 | 0 | 242 | 27 | 3 | 968 | 39 | 201 |
| G14-P01 | 297 | 618 | 4 | 69 | 0 | 12 | 528 | 83 | 143 |
| G15-P01 | 1 | 18 | 0 | 0 | 0 | 0 | 92 | 0 | 3 |
| G16-P01 | 78 | 1,043 | 15 | 22 | 0 | 31 | 904 | 2 | 559 |
| G17-P01 | 11 | 284 | 13 | 0 | 0 | 10 | 408 | 1 | 95 |
| G17-P02 | 452 | 395 | 16 | 29 | 2 | 19 | 392 | 20 | 266 |
| G18-P01 | 6 | 387 | 2 | 0 | 8 | 7 | 509 | 2 | 92 |
| G19-P01 | 427 | 410 | 21 | 31 | 0 | 17 | 226 | 0 | 109 |
| G19-P02 | 145 | 142 | 0 | 7 | 0 | 6 | 158 | 0 | 29 |
| G20-P01 | 123 | 971 | 3 | 9 | 0 | 32 | 921 | 19 | 503 |
| G21-P01 | 409 | 674 | 4 | 32 | 0 | 8 | 727 | 20 | 305 |
| G22-P01 | 86 | 430 | 5 | 7 | 17 | 10 | 231 | 2 | 195 |
| G22-P02 | 3,236 | 55 | 0 | 106 | 0 | 1 | 44 | 0 | 23 |
| G22-P03 | 58 | 236 | 1 | 5 | 0 | 3 | 286 | 0 | 130 |
| G22-P04 | 1,660 | 118 | 0 | 62 | 0 | 1 | 59 | 1 | 67 |
| G23-P01 | 82 | 74 | 0 | 19 | 30 | 1 | 144 | 180 | 68 |
| G23-P02 | 83 | 61 | 0 | 23 | 36 | 1 | 151 | 201 | 65 |
| G24-P01 | 31 | 24 | 1 | 5 | 0 | 1 | 25 | 1 | 15 |
| G25-P01 | 0 | 10 | 0 | 0 | 2 | 0 | 42 | 14 | 10 |
| G26-P01 | 20 | 4 | 0 | 1 | 0 | 0 | 21 | 0 | 5 |
| G27-P01 | 0 | 84 | 0 | 0 | 137 | 2 | 205 | 98 | 70 |
| G28-P01 | 89 | 50 | 0 | 3 | 154 | 0 | 150 | 62 | 53 |
| G29-P01 | 0 | 94 | 1 | 0 | 38 | 0 | 141 | 242 | 149 |
| G30-P01 | 2 | 15 | 0 | 0 | 163 | 0 | 62 | 237 | 130 |
| G30-P02 | 0 | 5 | 0 | 0 | 0 | 0 | 29 | 62 | 56 |
| G31-P01 | 0 | 29 | 0 | 0 | 39 | 1 | 78 | 92 | 80 |
| G31-P02 | 0 | 61 | 0 | 0 | 0 | 1 | 130 | 1 | 79 |
| G31-P03 | 0 | 18 | 0 | 0 | 22 | 0 | 105 | 120 | 104 |
| G32-P01 | 0 | 85 | 0 | 0 | 89 | 1 | 159 | 3 | 305 |
| G32-P02 | 0 | 33 | 0 | 0 | 235 | 1 | 89 | 98 | 97 |
| G32-P03 | 0 | 9 | 0 | 0 | 12 | 0 | 101 | 45 | 85 |
| G33-P01 | 145 | 2,930 | 32 | 25 | 0 | 105 | 631 | 2 | 108 |
| G34-P01 | 724 | 913 | 13 | 43 | 0 | 22 | 205 | 0 | 137 |
| G34-P02 | 224 | 1,887 | 32 | 28 | 0 | 94 | 171 | 0 | 275 |
| G34-P03 | 3 | 2,906 | 0 | 0 | 0 | 108 | 308 | 0 | 559 |
| G35-P01 | 1,951 | 363 | 5 | 75 | 0 | 12 | 368 | 0 | 75 |
| G35-P02 | 3,697 | 1,586 | 13 | 136 | 1 | 42 | 1,012 | 0 | 598 |
| G36-P01 | 0 | 8 | 0 | 0 | 9 | 0 | 25 | 37 | 38 |
| G37-P01 | 0 | 3 | 0 | 0 | 78 | 0 | 20 | 207 | 58 |
| G38-P01 | 0 | 1 | 0 | 0 | 0 | 0 | 61 | 357 | 44 |

NA: Not Available.

Note: The article G10-P02 was not found in Scopus. PlumX does not provided citation data from Scopus as well.

**Appendix D**

Source entity types

| N | Entity  type | Scope | Domain names | URL citations |
| --- | --- | --- | --- | --- |
| 1 | **Academic database** | Website offering access to an online bibliographic database (e.g., dimensions.ai). | 52 | 342 |
| 2 | **App** | Website oriented to offering a software to be downloaded or purchased (e.g., issuhub.com) | 14 | 80 |
| 3 | **Blogs provider** | Website offering blog services (e.g., wordpress.com) | 5 | 17 |
| 4 | **Clinic** | Website representing a clinic (e.g., wellnessdoctorrx.com). | 3 | 12 |
| 5 | **Codes Hub** | Website collecting code from different users, offering a search functionality (e.g., sydit.se). | 4 | 7 |
| 6 | **Company** | Website representing a private firm (e.g., wire-technology.com). Publishers and media are excluded. | 91 | 629 |
| 7 | **Directory** | Website offering a list of URLs pointing to specific thematic websites (e.g., best-university.com). | 38 | 74 |
| 8 | **Encyclopaedia** | Website offering one or more encyclopaedias entries (e.g., wikipedia.org). | 46 | 774 |
| 9 | **Event** | Website informing about one specific activity (e.g., celebratingbletchleypark.co.uk). | 2 | 6 |
| 10 | **Gambling** | Website oriented to online game (e.g., online casinos). | 6 | 11 |
| 11 | **Governmental body** | Website representing any official administration or service, not related with health (e.g., csiro.au). | 1 | 3 |
| 12 | **Health government body** | Website representing an official health-related government entity, which can include research-related organizations (e.g., nih.gov). | 15 | 107 |
| 13 | **Health information hub** | Website including a collection of posts related with health (e.g., outsourcing-pharma.com). | 71 | 335 |
| 14 | **Higher Education Institution** | Website representing a university or any other educational body (e.g., columbia.edu). | 83 | 2,326 |
| 15 | **Hospital** | Website representing a hospital (e.g., usz.ch). | 5 | 5 |
| 16 | **Images Hub** | Website offering a collection of pictures (e.g., nodexlgraphgallery.org). |  |  |
| 17 | **Library** | Website representing a Library, regardless the institution where the library is embedded (e.g., dntb.gov.ua). | 2 | 2 |
| 18 | **News Hub** | Website offering a collection of posts and news including a wide variety of topics and themes (e.g., wopular.com). | 1 | 1 |
| 19 | **Non-Academic database** | Website offering access to an online database unrelated with scientific and academic literature. | 64 | 142 |
| 20 | **Online Fora** | Websites offering forum services to users (e.g., stackexchange.com). | 30 | 75 |
| 21 | **Organization** | Websites representing any type of Association, Federation, co-op, excluding research organizations (e.g., equator-network.org). | 7 | 12 |
| 22 | **Other information hubs** | Websites or blogs offering a collection of posts in one specific topic, excluding health topics (e.g., ephorie.de). | 95 | 432 |
| 23 | **Parked** | Websites whose web domain is registered but no web-content has been included yet. | 52 | 196 |
| 24 | **Personal website** | Website representing an individual (e.g., nerler.com). | 87 | 1,559 |
| 25 | **Porn** | Websites including adult content and explicit sex. | 6 | 6 |
| 26 | **Promos** | Websites including promotional events and coupons. | 2 | 2 |
| 27 | **Publisher** | Websites representing a publisher, including books and journals (e.g., springer.com). | 222 | 10,169 |
| 28 | **Research centre** | Website representing a research centre, regardless the status (public or private) and the parent institution (e.g., ktdrr.org). | 7 | 11 |
| 29 | **Research group** | Website representing a research group, regardless the status (public or private) and the parent institution (e.g., https://www.pocog.org.au). | 6 | 17 |
| 30 | **Research institute** | Website representing a research institute, regardless the status (public or private) and the parent institution (e.g., sahmriresearch.org). | 3 | 25 |
| 31 | **Research project** | Website representing a research project, regardless the funding body (e.g., humanbehaviourchange.org). | 3 | 4 |
| 32 | **Science Hub** | Website including a collection of posts related with scientific content (e.g., the-scientist.com). | 8 | 12 |
| 33 | **Search engine** | Website offering a search engine (e.g., zapmeta.ng). | 4 | 14 |
| 34 | **Shopping** | Website offering goods and products to be online purchased through an ecommerce functionality (e.g., popflock.com). | 10 | 52 |
| 35 | **Social Networking Site** | Website offering a social media functionality, either scientific-related (e.g., figshare.com) or non-scientific (e.g., scwacy.com). | 1 | 1 |
| 36 | **Tourism** | Website offering touristic information about a city, region or country (e.g., irvinescotland.info). | 4 | 20 |
| 37 | **Unknown** | Websites whose contents have not been possible to be classified, either because its content is not accessible or because it could not be determined with precision. | 6,987 | 66,272 |
| 38 | **Videos Hub** | Website offering a collection of videos, including a wide variety of topics and themes (e.g., wikivisually.com). | 2 | 18 |

**Appendix E**

Number of linking domain names per entity type for each article

| Publication | Publisher | Organization | Company | Higher Education  Institution | Health information  hub | News  Hub | Other  information hubs | Academic  database | Encyclopedia | Directory | Other | All |
| --- | --- | --- | --- | --- | --- | --- | --- | --- | --- | --- | --- | --- |
| G01-p1 | 24 | 6 | 1 | 8 | 1 | 0 | 1 | 2 | 0 | 4 | 8 | 55 |
| G02-p1 | 45 | 21 | 10 | 18 | 21 | 0 | 0 | 3 | 0 | 1 | 8 | 127 |
| G03-p1 | 17 | 1 | 0 | 5 | 0 | 0 | 0 | 0 | 0 | 8 | 3 | 34 |
| G04-p1 | 22 | 0 | 1 | 16 | 7 | 2 | 3 | 2 | 0 | 0 | 1 | 54 |
| G05-p1 | 28 | 0 | 1 | 0 | 0 | 0 | 0 | 0 | 0 | 1 | 0 | 30 |
| G06-p1 | 371 | 2 | 15 | 53 | 2 | 0 | 0 | 9 | 0 | 0 | 4 | 456 |
| G06-p10 | 261 | 0 | 4 | 68 | 0 | 0 | 0 | 8 | 1 | 0 | 2 | 344 |
| G06-p2 | 589 | 7 | 5 | 99 | 15 | 0 | 1 | 12 | 0 | 0 | 10 | 738 |
| G06-p3 | 883 | 18 | 64 | 164 | 64 | 18 | 17 | 24 | 290 | 2 | 37 | 1,581 |
| G06-p4 | 128 | 0 | 2 | 17 | 0 | 0 | 0 | 4 | 0 | 0 | 4 | 155 |
| G06-p5 | 51 | 0 | 0 | 4 | 0 | 0 | 0 | 5 | 0 | 0 | 2 | 62 |
| G06-p6 | 395 | 3 | 1 | 28 | 0 | 0 | 0 | 1 | 0 | 0 | 5 | 433 |
| G06-p7 | 209 | 10 | 1 | 83 | 2 | 0 | 1 | 10 | 0 | 0 | 18 | 334 |
| G06-p8 | 9 | 2 | 2 | 1 | 0 | 0 | 0 | 0 | 0 | 0 | 0 | 14 |
| G06-p9 | 706 | 11 | 43 | 144 | 37 | 17 | 10 | 18 | 188 | 5 | 32 | 1211 |
| G07-p1 | 983 | 6 | 9 | 201 | 2 | 0 | 0 | 5 | 1 | 2 | 5 | 1,,214 |
| G07-p2 | 723 | 3 | 8 | 183 | 8 | 2 | 0 | 12 | 18 | 12 | 7 | 976 |
| G08-p1 | 174 | 1 | 6 | 32 | 2 | 0 | 0 | 10 | 0 | 1 | 5 | 231 |
| G08-p2 | 179 | 0 | 9 | 37 | 0 | 0 | 4 | 8 | 7 | 0 | 5 | 249 |
| G09-p1 | 7 | 0 | 0 | 1 | 0 | 0 | 0 | 1 | 0 | 2 | 2 | 13 |
| G10-p1 | 12 | 1 | 1 | 2 | 1 | 2 | 0 | 0 | 0 | 0 | 5 | 24 |
| G10-p2 | 12 | 0 | 0 | 1 | 0 | 0 | 0 | 0 | 0 | 0 | 0 | 13 |
| G11-p1 | 711 | 19 | 20 | 121 | 2 | 3 | 0 | 31 | 1 | 9 | 18 | 935 |
| G12-p1 | 171 | 8 | 18 | 29 | 0 | 8 | 9 | 3 | 55 | 0 | 18 | 319 |
| G13-p1 | 157 | 0 | 2 | 32 | 0 | 0 | 0 | 3 | 0 | 2 | 5 | 201 |
| G14-p1 | 119 | 4 | 3 | 11 | 1 | 0 | 0 | 0 | 0 | 0 | 5 | 143 |
| G15-p1 | 2 | 0 | 0 | 0 | 0 | 0 | 0 | 0 | 0 | 0 | 1 | 3 |
| G16-p1 | 334 | 9 | 22 | 87 | 15 | 3 | 5 | 4 | 68 | 0 | 12 | 559 |
| G17-p1 | 79 | 0 | 2 | 11 | 0 | 0 | 0 | 0 | 0 | 0 | 3 | 95 |
| G17-p2 | 110 | 7 | 26 | 22 | 7 | 3 | 21 | 1 | 49 | 0 | 20 | 266 |
| G18-p1 | 72 | 4 | 5 | 6 | 0 | 0 | 0 | 4 | 0 | 0 | 1 | 92 |
| G19-p1 | 85 | 4 | 1 | 16 | 0 | 0 | 0 | 2 | 0 | 0 | 1 | 109 |
| G19-p2 | 24 | 2 | 0 | 3 | 0 | 0 | 0 | 0 | 0 | 0 | 0 | 29 |
| G20-p1 | 354 | 1 | 12 | 80 | 10 | 2 | 4 | 6 | 26 | 0 | 8 | 503 |
| G21-p1 | 140 | 3 | 0 | 155 | 3 | 1 | 0 | 1 | 0 | 0 | 2 | 305 |
| G22-p1 | 163 | 1 | 4 | 15 | 4 | 0 | 0 | 6 | 0 | 0 | 2 | 195 |
| G22-p2 | 19 | 0 | 0 | 3 | 0 | 0 | 0 | 0 | 0 | 0 | 1 | 23 |
| G22-p3 | 110 | 0 | 7 | 7 | 0 | 0 | 0 | 4 | 0 | 0 | 2 | 130 |
| G22-p4 | 59 | 1 | 2 | 3 | 0 | 0 | 0 | 2 | 0 | 0 | 0 | 67 |
| G23-p1 | 20 | 10 | 15 | 19 | 0 | 0 | 1 | 2 | 0 | 0 | 1 | 68 |
| G23-p2 | 11 | 9 | 12 | 22 | 1 | 0 | 1 | 5 | 0 | 0 | 4 | 65 |
| G24-p1 | 9 | 0 | 0 | 0 | 0 | 0 | 0 | 5 | 0 | 0 | 1 | 15 |
| G25-p1 | 5 | 1 | 0 | 0 | 0 | 0 | 0 | 3 | 0 | 1 | 0 | 10 |
| G26-p1 | 3 | 0 | 0 | 0 | 0 | 0 | 0 | 0 | 0 | 1 | 1 | 5 |
| G27-p1 | 30 | 1 | 7 | 2 | 0 | 4 | 10 | 3 | 0 | 2 | 11 | 70 |
| G28-p1 | 11 | 8 | 2 | 18 | 9 | 2 | 0 | 1 | 0 | 0 | 2 | 53 |
| G29-p1 | 40 | 2 | 7 | 63 | 1 | 3 | 5 | 5 | 0 | 0 | 23 | 149 |
| G30-p1 | 5 | 31 | 9 | 20 | 0 | 6 | 13 | 17 | 0 | 0 | 29 | 130 |
| G30-p2 | 3 | 28 | 0 | 8 | 9 | 0 | 0 | 7 | 0 | 0 | 1 | 56 |
| G31-p1 | 17 | 6 | 5 | 6 | 13 | 3 | 6 | 0 | 0 | 5 | 19 | 80 |
| G31-p2 | 12 | 10 | 9 | 10 | 5 | 3 | 1 | 15 | 0 | 0 | 14 | 79 |
| G31-p3 | 4 | 10 | 17 | 7 | 4 | 16 | 22 | 2 | 0 | 2 | 20 | 104 |
| G32-p1 | 19 | 39 | 81 | 6 | 30 | 36 | 51 | 20 | 0 | 3 | 20 | 305 |
| G32-p2 | 15 | 28 | 21 | 4 | 14 | 1 | 1 | 0 | 0 | 1 | 12 | 97 |
| G32-p3 | 2 | 31 | 24 | 3 | 6 | 3 | 2 | 0 | 0 | 3 | 11 | 85 |
| G33-p1 | 76 | 3 | 0 | 0 | 7 | 1 | 0 | 4 | 7 | 0 | 10 | 108 |
| G34-p1 | 107 | 3 | 1 | 21 | 0 | 0 | 0 | 3 | 0 | 0 | 2 | 137 |
| G34-p2 | 201 | 2 | 2 | 62 | 0 | 0 | 0 | 4 | 0 | 0 | 4 | 275 |
| G34-p3 | 425 | 1 | 6 | 107 | 0 | 0 | 0 | 8 | 8 | 0 | 4 | 559 |
| G35-p1 | 19 | 0 | 11 | 3 | 6 | 2 | 2 | 1 | 25 | 0 | 6 | 75 |
| G35-p2 | 424 | 2 | 16 | 111 | 6 | 2 | 2 | 3 | 26 | 0 | 6 | 598 |
| G36-p1 | 2 | 5 | 9 | 13 | 1 | 2 | 0 | 3 | 1 | 0 | 2 | 38 |
| G37-p1 | 3 | 27 | 0 | 4 | 1 | 1 | 1 | 16 | 0 | 2 | 3 | 58 |
| G38-p1 | 2 | 6 | 1 | 8 | 4 | 2 | 1 | 10 | 0 | 3 | 7 | 44 |

**Appendix F**

Object genres

| Object genre | Scope |
| --- | --- |
| Author guidelines | Publishing recommendations and guidelines for authors. |
| Bibliographic record | Machine-readable record created by a bibliographic database (e.g., repository) which includes bibliographic metadata. This record usually corresponds to the guideline publication itself. |
| Comment / Forum | Content published as part of an online forum post. |
| Data Report / Altmetric data provider | Data sheet automatically created by an altmetrics data provider. |
| Data report / SEO tool | Data sheet automatically created by SEO tools. |
| Institutional information | Descriptive content related to institutional information. |
| News | News published in different website categories (e.g., (media, thematic information, professional blogs) |
| Not found | Non-accessible webpages |
| Personal page | Personal webpages including academic CVs, that include in turn bibliographic references. |
| Post | Periodic publication included in multidisciplinary websites. |
| Post / Tweet | A micro-post published in the social networking site Twitter. |
| Publication / Article | Scientific publication (in this case a journal article). The link can be placed in any section of the publication. |
| Publication / Book | Scientific publication (including a thesis). The link can be placed in any section of the publication. |
| Publication / Mirror | Scientific publication hosted in a publisher’s mirror. |
| Publication / Summary | A summary of one publication (necessarily not the abstract) published in an academic website. |
| References list | Set of references gathered by bibliographic databases, repositories, wikis or institutional websites (e.g., research centers, research groups). |
| Resource list | Directory of thematic web resources. |
| Teaching material | Contents created to support teaching (e.g., syllabi, notes) |
| Undetermined | Accessible webpages without links |
| Void | Non-accessible webpages. |
| Wiki entry | Articles published in a collaboratively developed website (e.g., Wikipedia). |

**Author guidelines**

Reporting publication cited: G17-P01

Source URL

<https://www.elsevier.com/journals/journal-of-emergency-nursing/0099-1767/guide-for-authors>

Target URL :

<https://www.thelancet.com/journals/lancet/article/PIIS0140-6736(07)61835-2/fulltext>


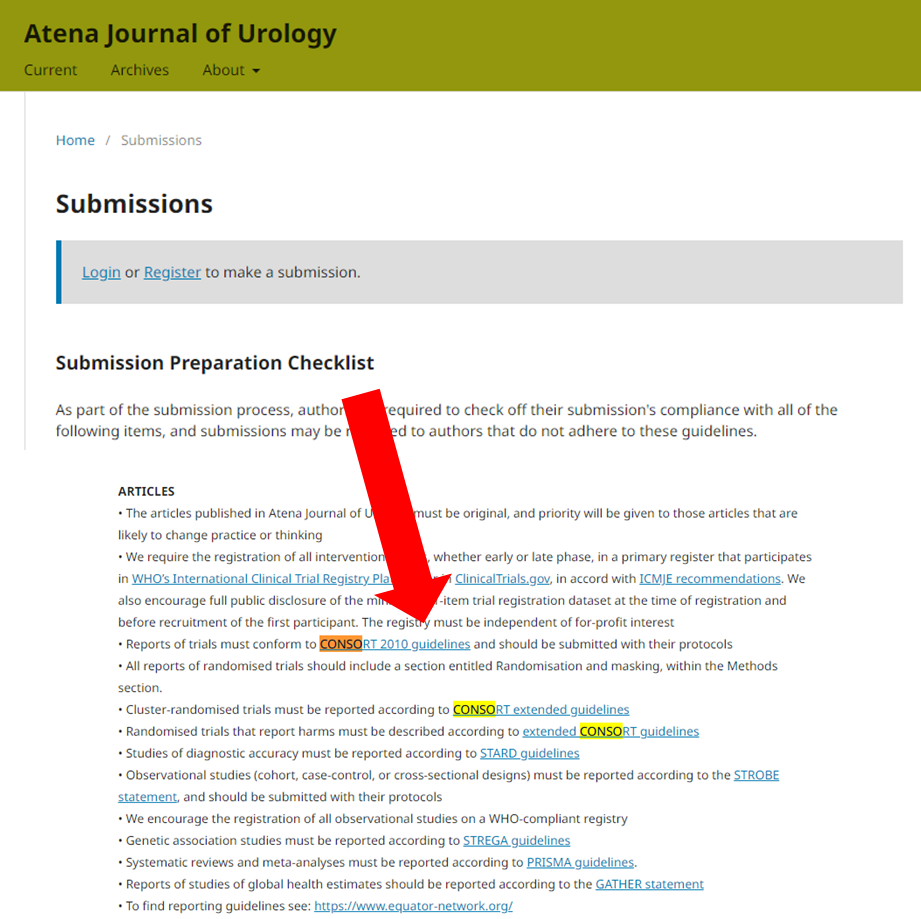


**Bibliographic record**

Reporting publication cited: G8-P02

Source URL:

<https://aura.abdn.ac.uk/handle/2164/7614>

Target URL:

<https://doi.org/10.1136/bmj.i5239>


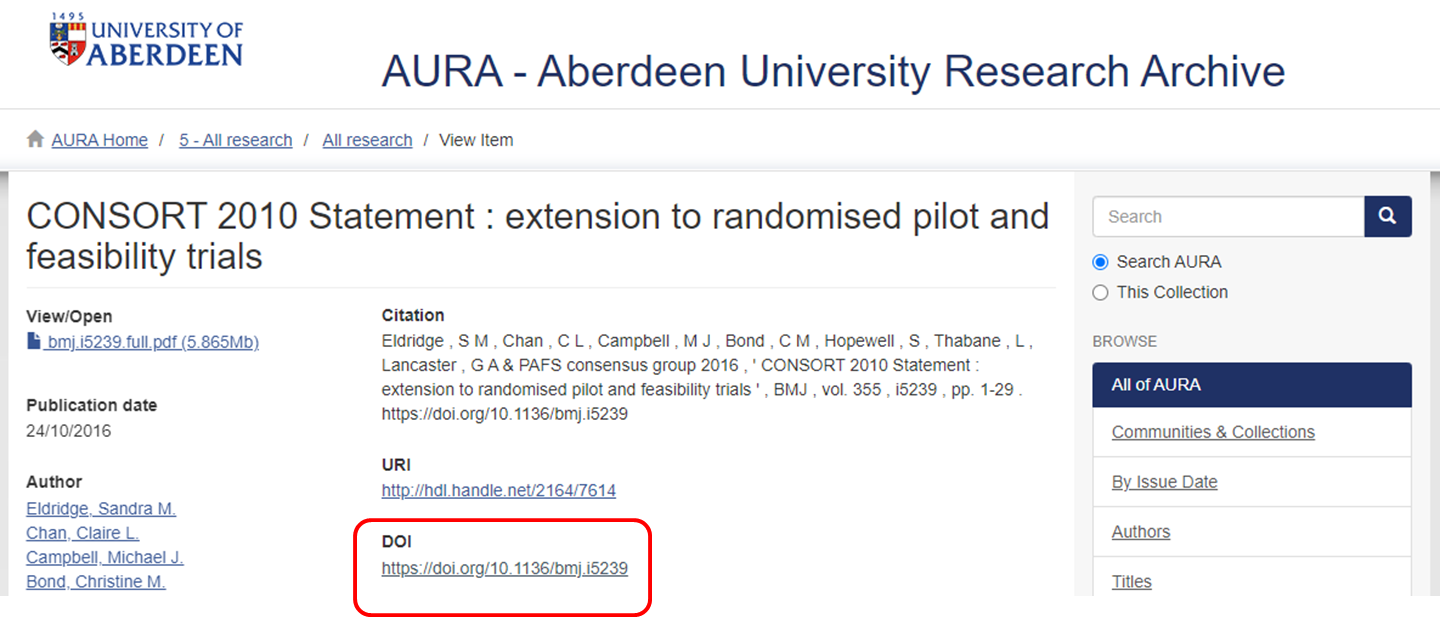


**Comment / Forum**

Reporting publication cited: G8-P02

Source URL:

<https://discourse.datamethods.org/t/reference-collection-to-push-back-against-common-statistical-myths/1787>

Target URL:

https://www.bmj.com/content/355/bmj.i5239


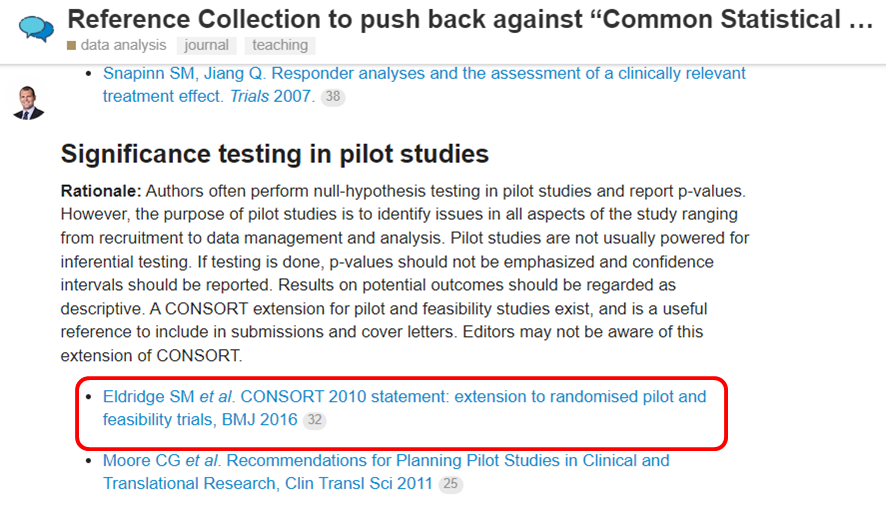


**Data report / Altmetric bibliographic report**

Reporting publication cited: G11-P01

Source URL:

<https://altmetric.com/details/2171807>

Target URL:

<https://doi.org/10.1136/bmj.g1687>


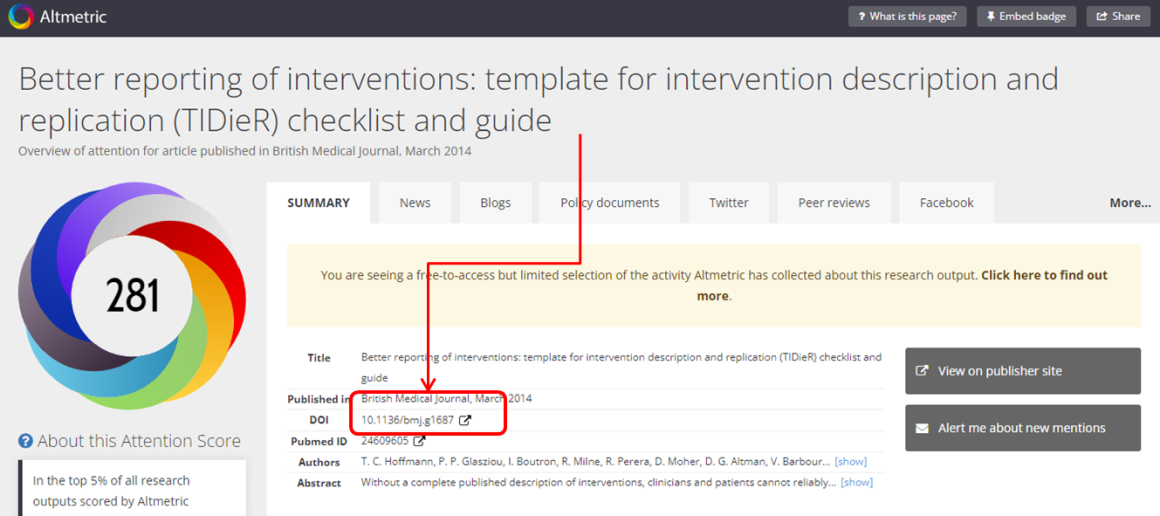


**Data report / SEO tool**

Reporting publication cited: G6-P09.

Source URL:

<https://bestref.net/doi/10.1136/bmj.c869>

Target URL:

<https://doi.org/10.1136/bmj.c869>


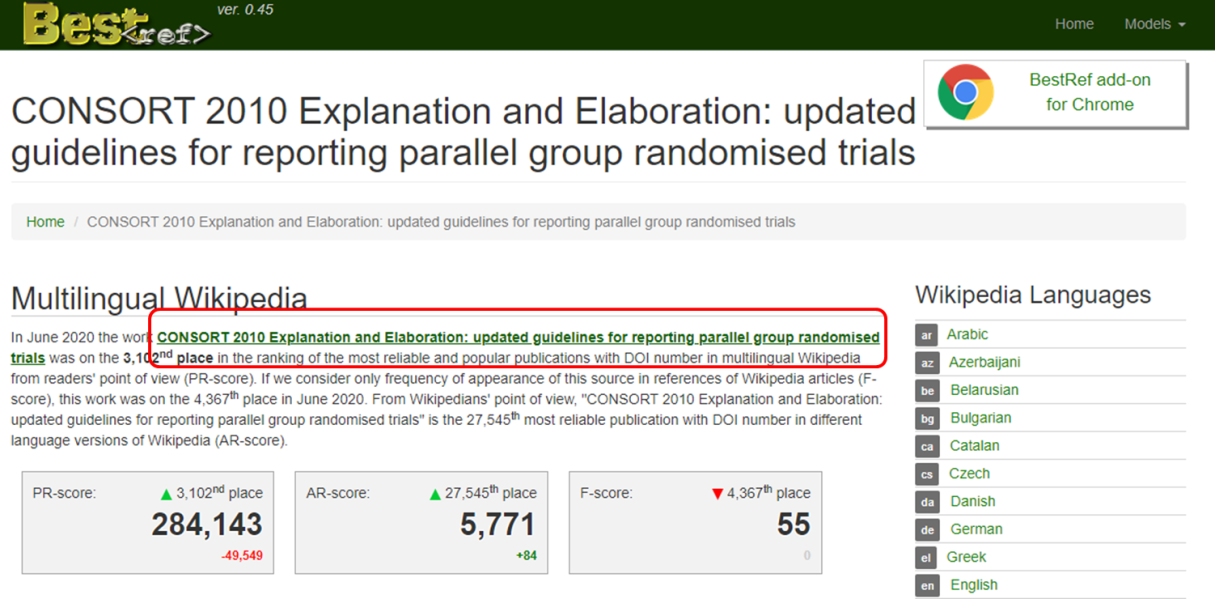


**Institutional information**

Reporting publication cited: G2-P01

Source URL:

<https://bristol.ac.uk/population-health-sciences/centres/surgical-research/research/outcome-measures/measures.html>

Target URL:

<https://jamanetwork.com/journals/jama/fullarticle/2671472>


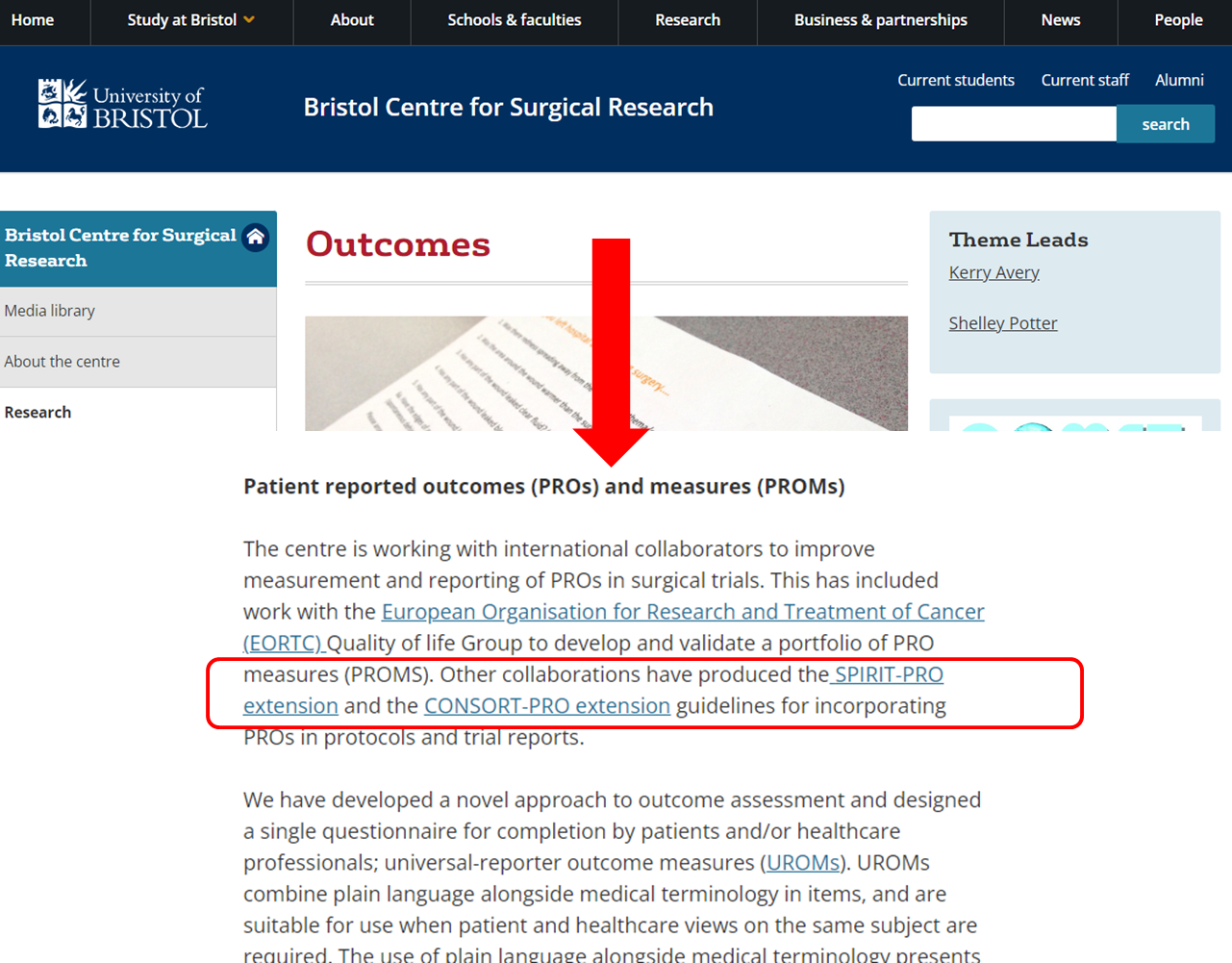


**News**

Reporting publication cited: G32-P03

Source URL :

<https://www.news-medical.net/news/20200910/New-guidelines-can-boost-transparency-of-clinical-trials-evaluating-AI-health-solutions.aspx>

Target URL :

<https://doi.org/10.1016/S2589-7500(20)30218-1>


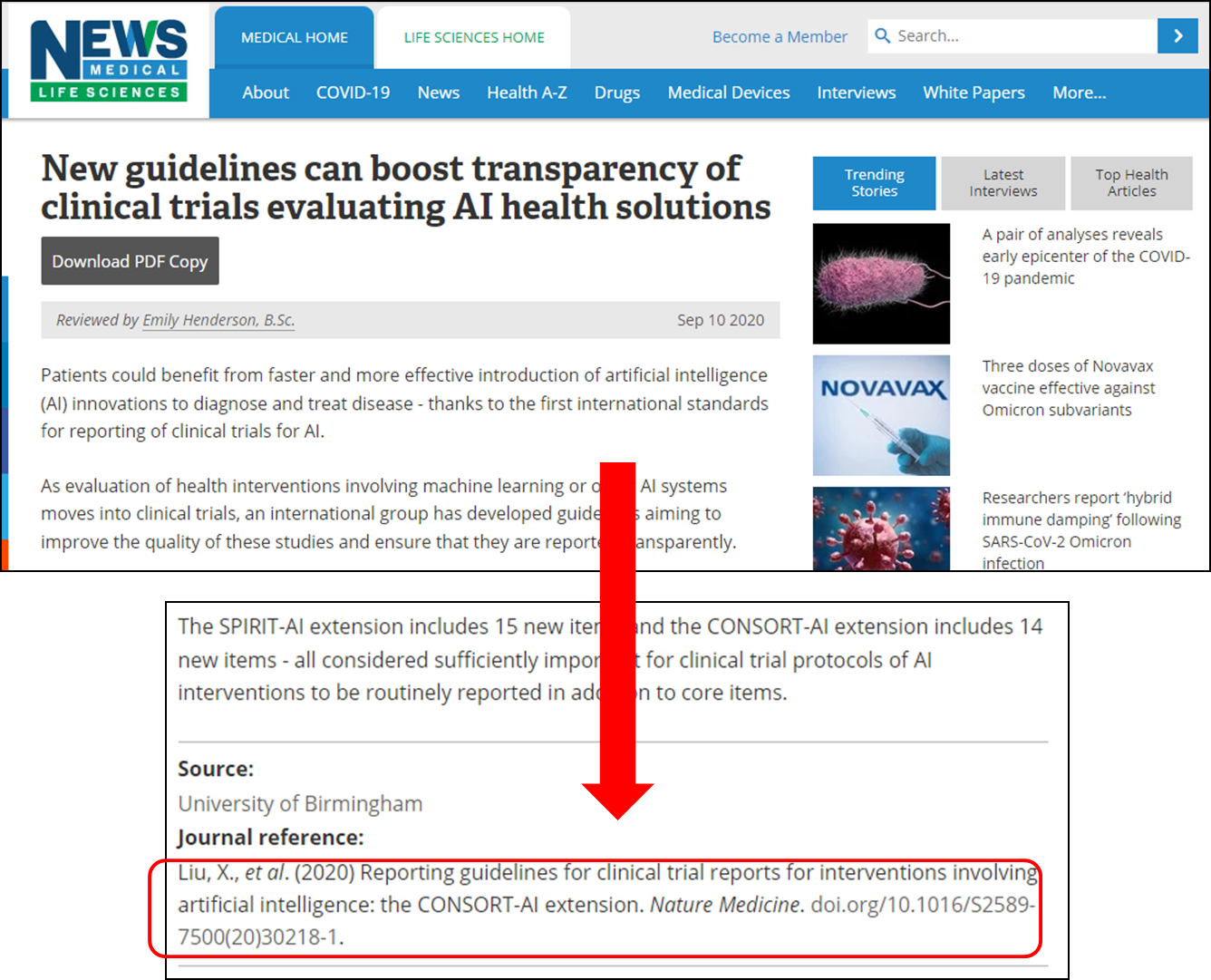


**Personal page**

Reporting publication: G23-P01, G23-P02

Source URL:

<https://www.birmingham.ac.uk/staff/profiles/social-policy/montgomery-paul.aspx>

Target URL:

<https://doi.org/10.1186/s13063-018-2733-1>


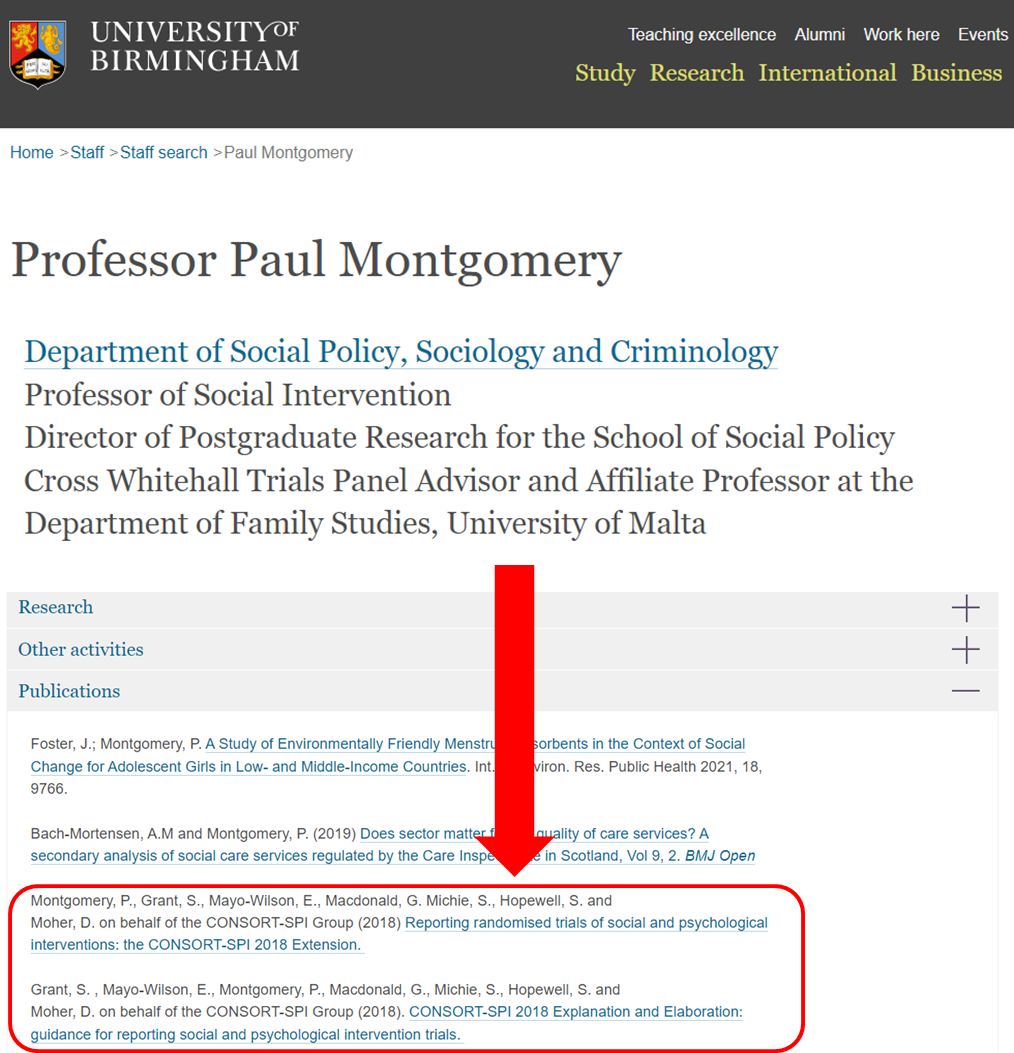


**Post**

Reporting publications cited: G6-P01, G6-P02, G6-P05, G6-P06, G6-P07, G6-P11

Source URL :

<https://cambridgemedicallibrary.blogspot.com/2010/03/new-consort-guidelines-for-rcts.html>

Target URL :

<http://www.annals.org/content/early/2010/03/18/0003-4819-152-11-201006010-00232.full?aimhp>

<http://www.biomedcentral.com/1741-7015/8/18/abstract>

<http://journals.lww.com/greenjournal/documents/CONSORT2010_ObstetGynecol.pdf>

<http://www.openmedicine.ca/article/view/352/315>

<http://www.plosmedicine.org/article/info%3Adoi%2F10.1371%2Fjournal.pmed.1000251>

<http://www.trialsjournal.com/content/11/1/32>


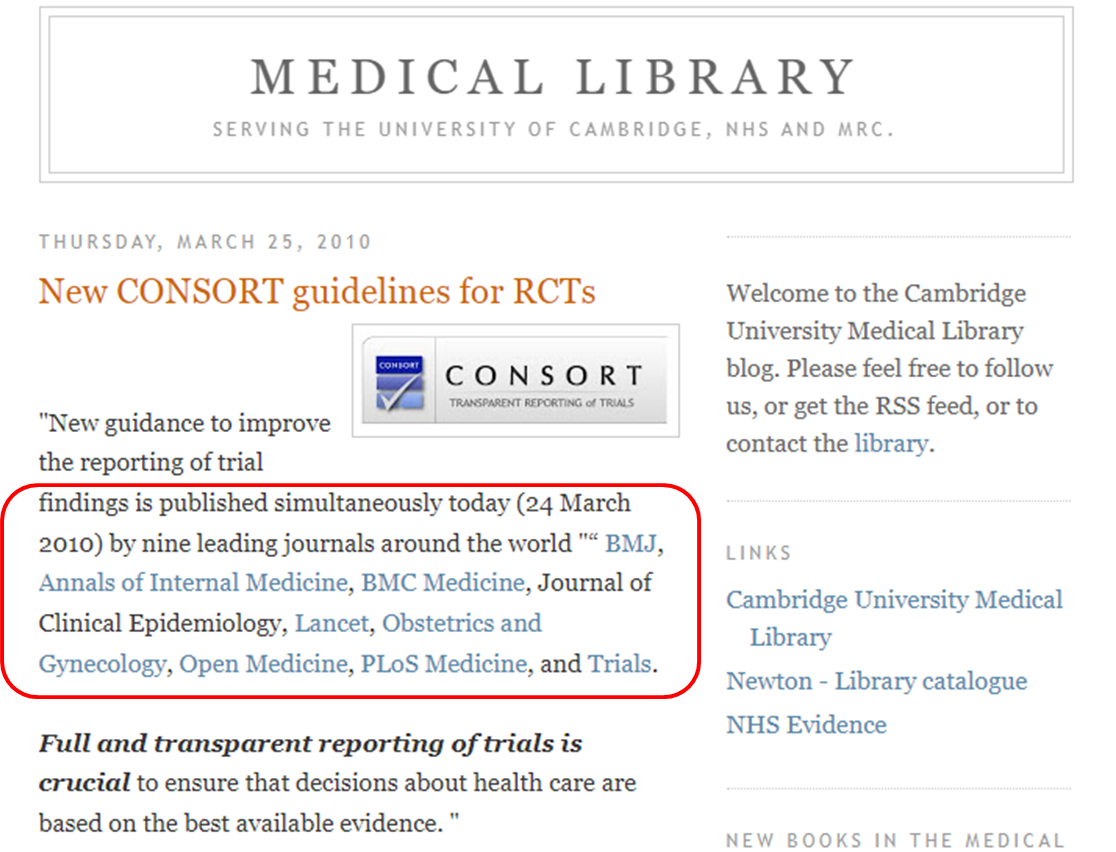


**Post / Tweet**

Reporting publications cited: G32-P01, G31-P01

Source URL :

<https://threadreaderapp.com/thread/1303727942153928705.html>

Target URLs :

<https://www.nature.com/articles/s41591-020-1037-7>

<https://doi.org/10.1016/S2589-7500(20)30219-3>

<http://www.bmj.com/content/370/bmj.m3210>

<https://www.nature.com/articles/s41591-020-1034-x>

<https://doi.org/10.1016/S2589-7500(20)30218-1>


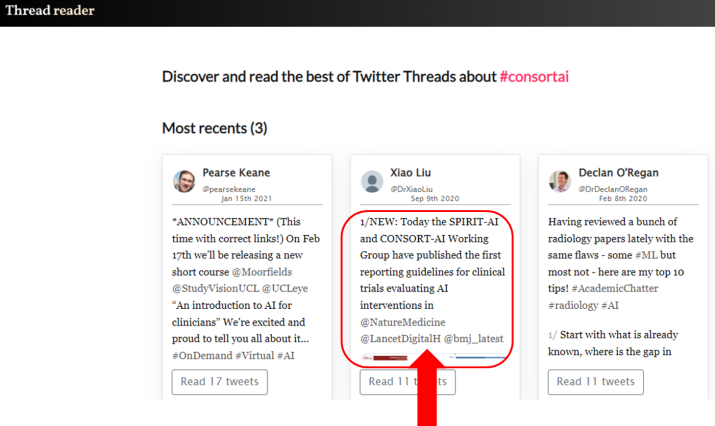


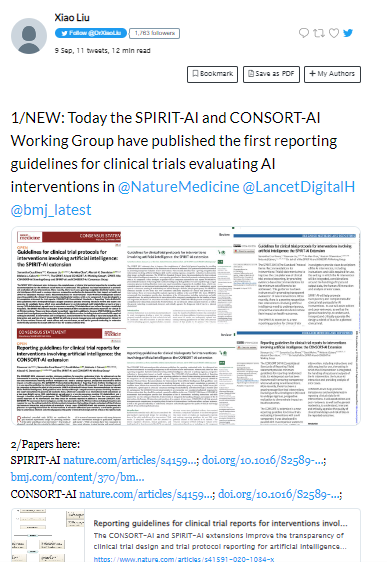


**Publication / Article**

Reporting publication cited: G11-P01

Source URL:

<https://idpjournal.biomedcentral.com/articles/10.1186/s40249-018-0474-8>

Target URL:

<https://www.ncbi.nlm.nih.gov/pubmed/24609605>

**
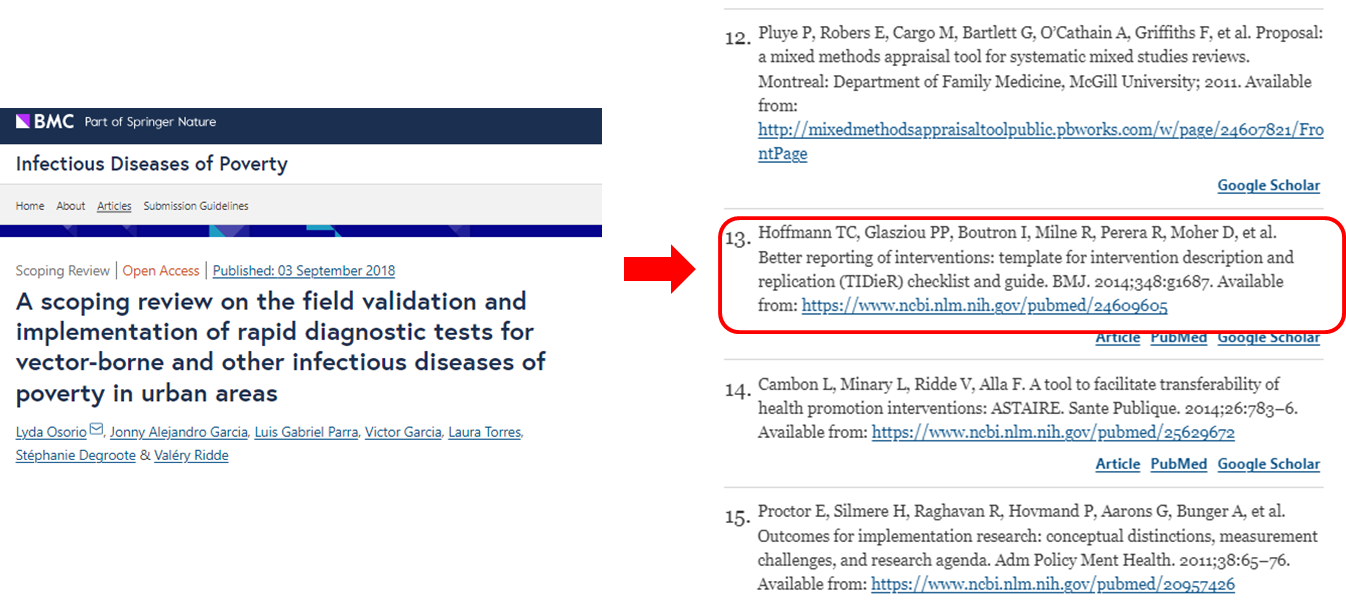
**

**Publication / Book**

Reporting publication cited: G17-P02

Source URL:

<https://deemagclinic.com/2020/12/03/art-of-thesis-writing/>

Target URL:

<https://doi.org/10.1371/journal.pmed.0050020>


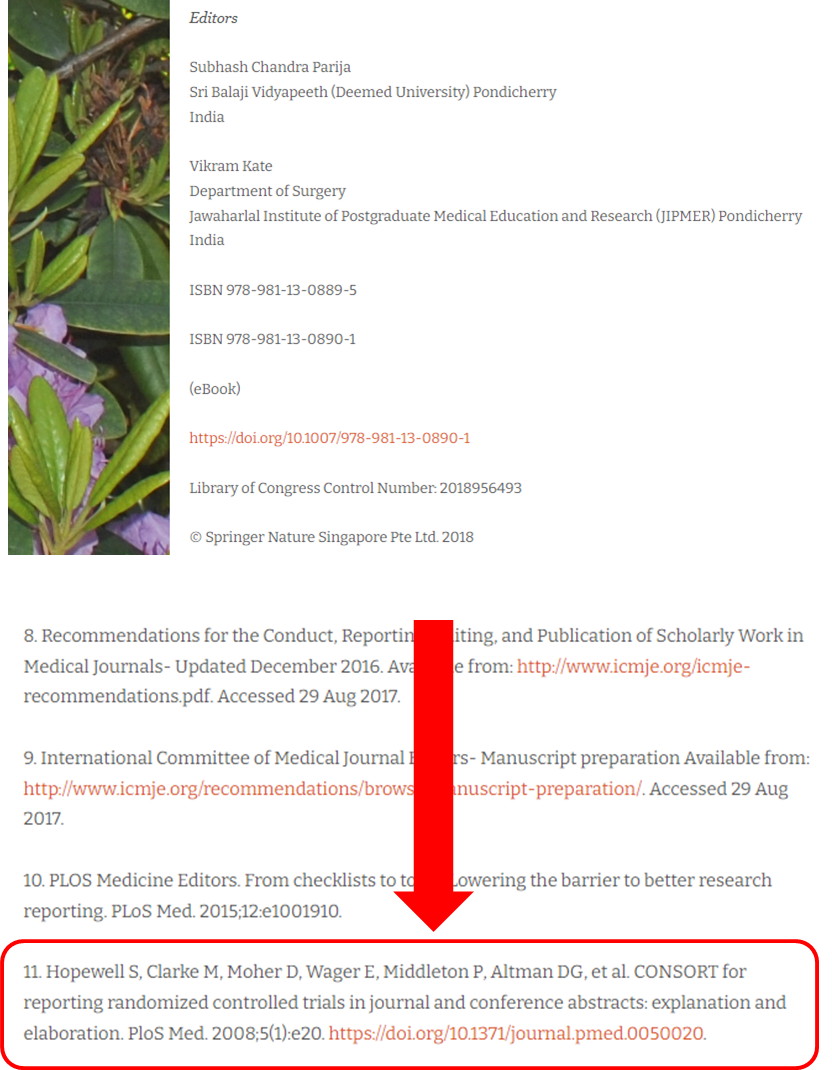


**Publication / Mirror**

Reporting publication cited: G9-P01

Source URL:

<https://0-advancesinsimulation-biomedcentral-com.brum.beds.ac.uk/articles/10.1186/s41077-017-0039-0>

Target URL:

<https://journals.lww.com/simulationinhealthcare/Fulltext/2016/08000/Reporting_Guidelines_for_Health_Care_Simulation.4.aspx>


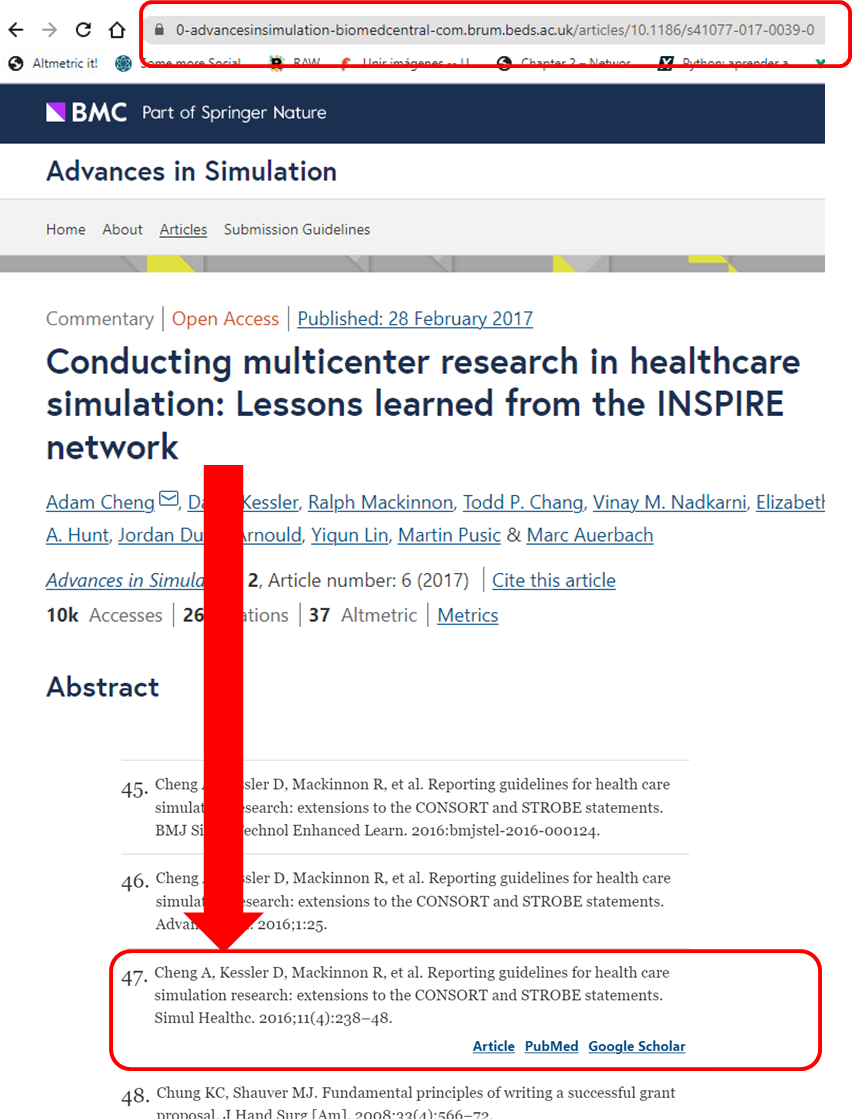


**Publication / Summary**

Reporting publication cited: G2-P01

Source URL:

<https://www.birmingham.ac.uk/research/cpror/research/spirit-pro.aspx>

Target URL:

<https://jamanetwork.com/journals/jama/fullarticle/2671472>


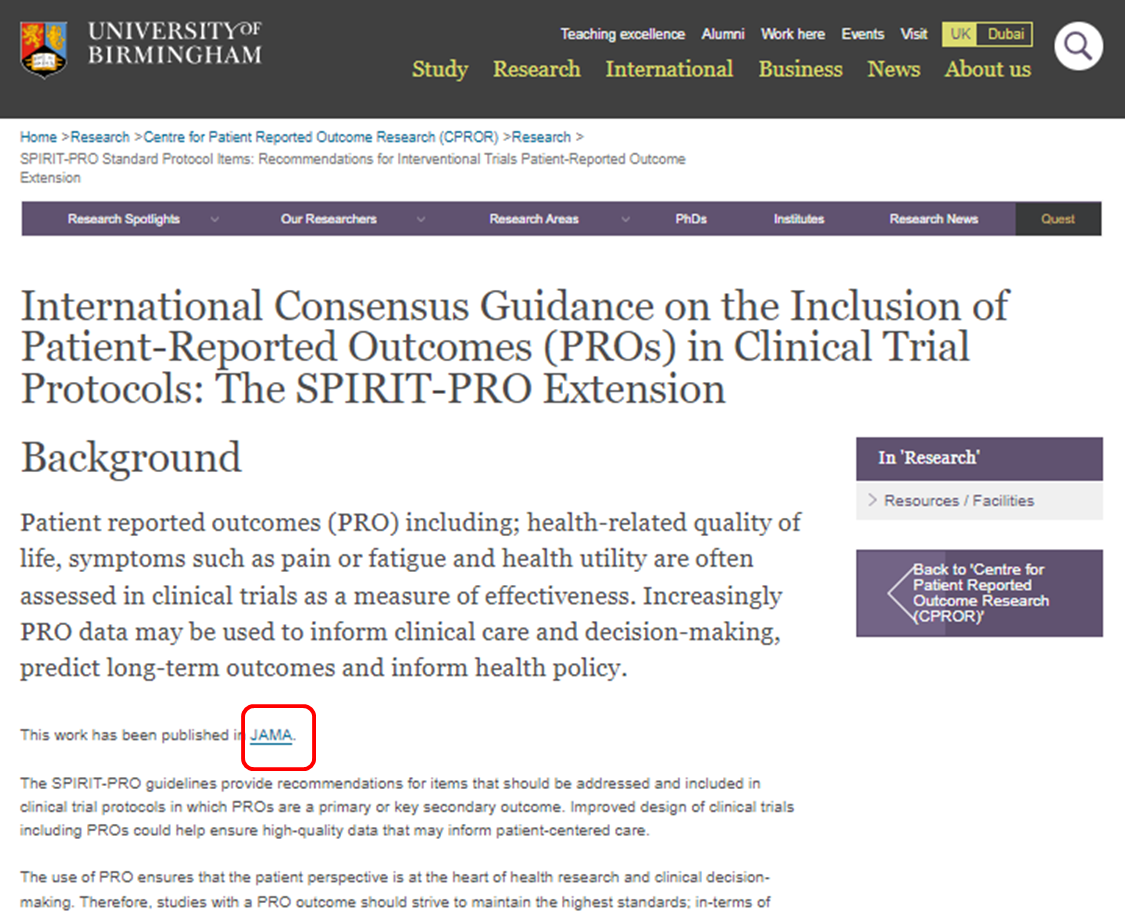


**References list**

Reporting publication cited: G31-P02 and G32-P01

Source URL:

<https://www.birmingham.ac.uk/research/applied-health/key%20publications.aspx>

Target URL:

<https://pubmed.ncbi.nlm.nih.gov/32908283/>

<https://pubmed.ncbi.nlm.nih.gov/32908284/>


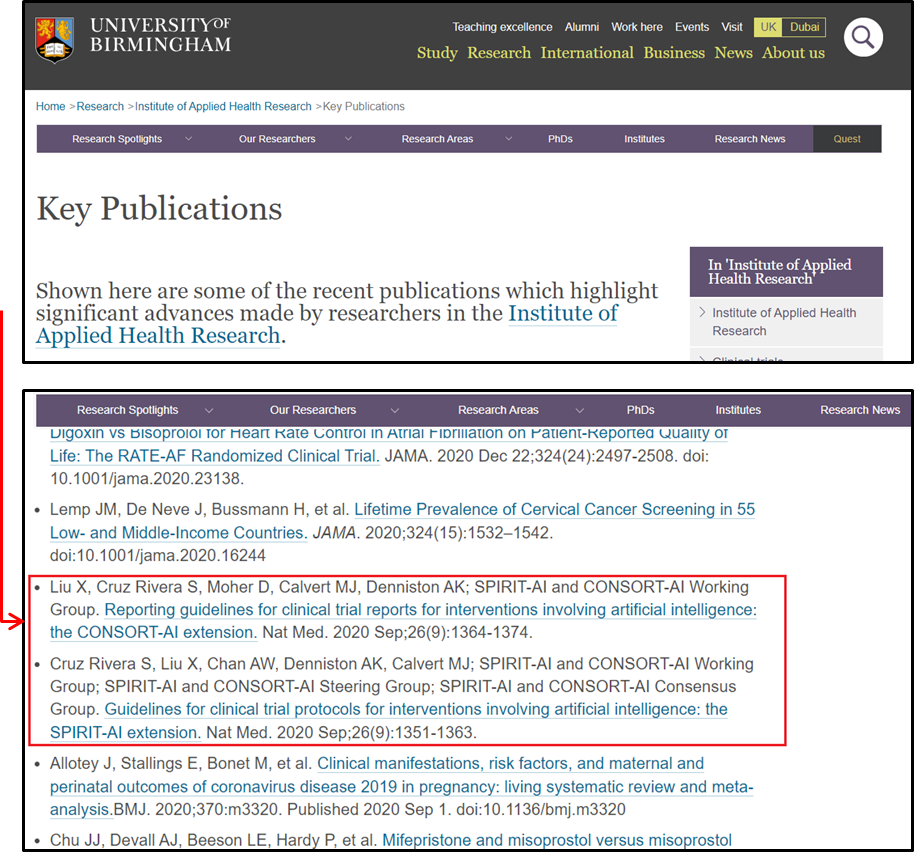


**Resource list**

Reporting publication cited: G38-P01

Source URL:

<https://ecrin.org/other-covid-19-resources>

Target URL:

<https://jamanetwork.com/journals/jama/fullarticle/2781397>


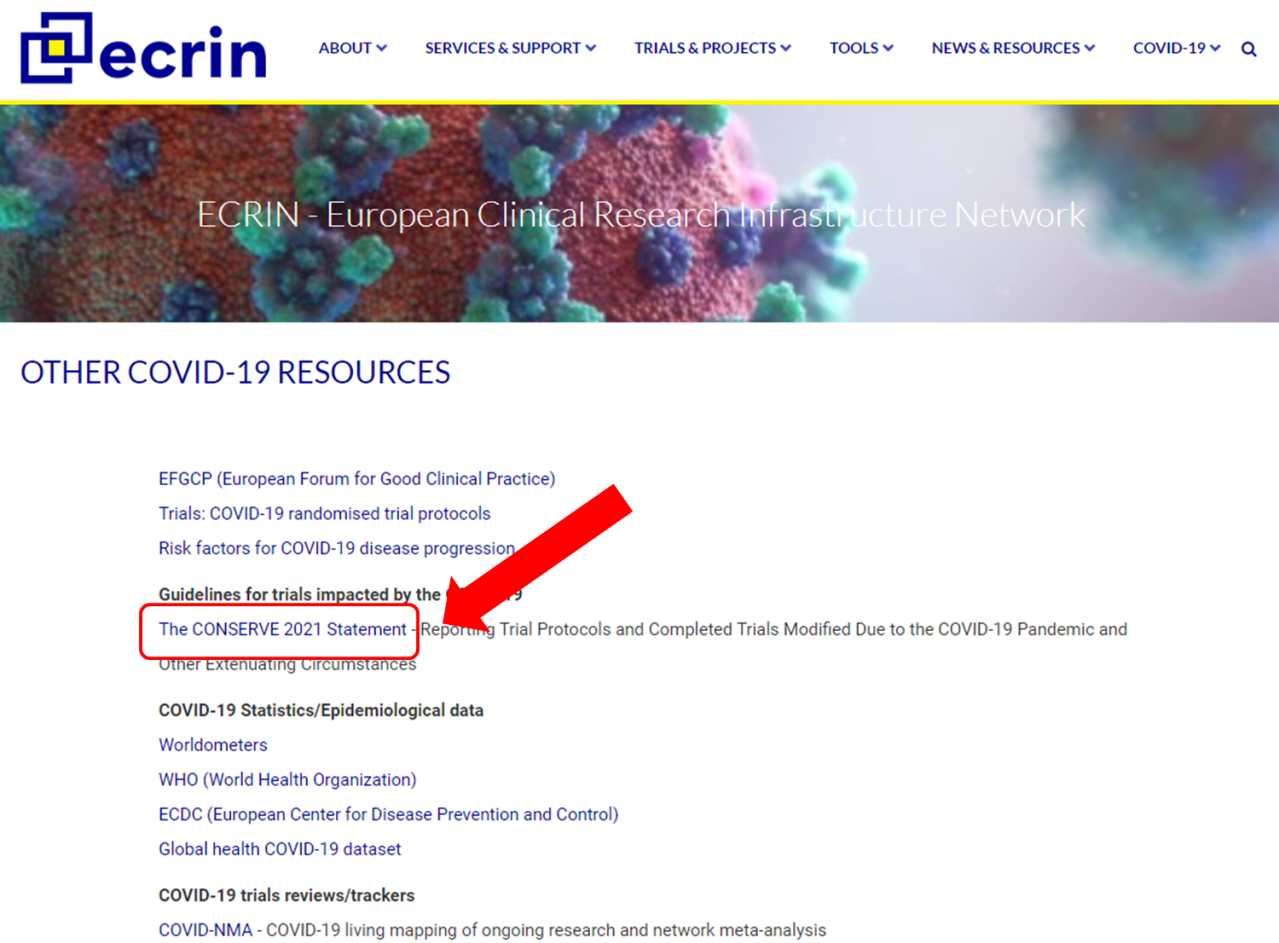


**Teaching material**

Reporting publication cited: G6-P01

Source URL:

<https://online.stat.psu.edu/stat509/lesson/13/13.4>

Target URL:

<https://www.acpjournals.org/doi/10.7326/0003-4819-152-11-201006010-00232>


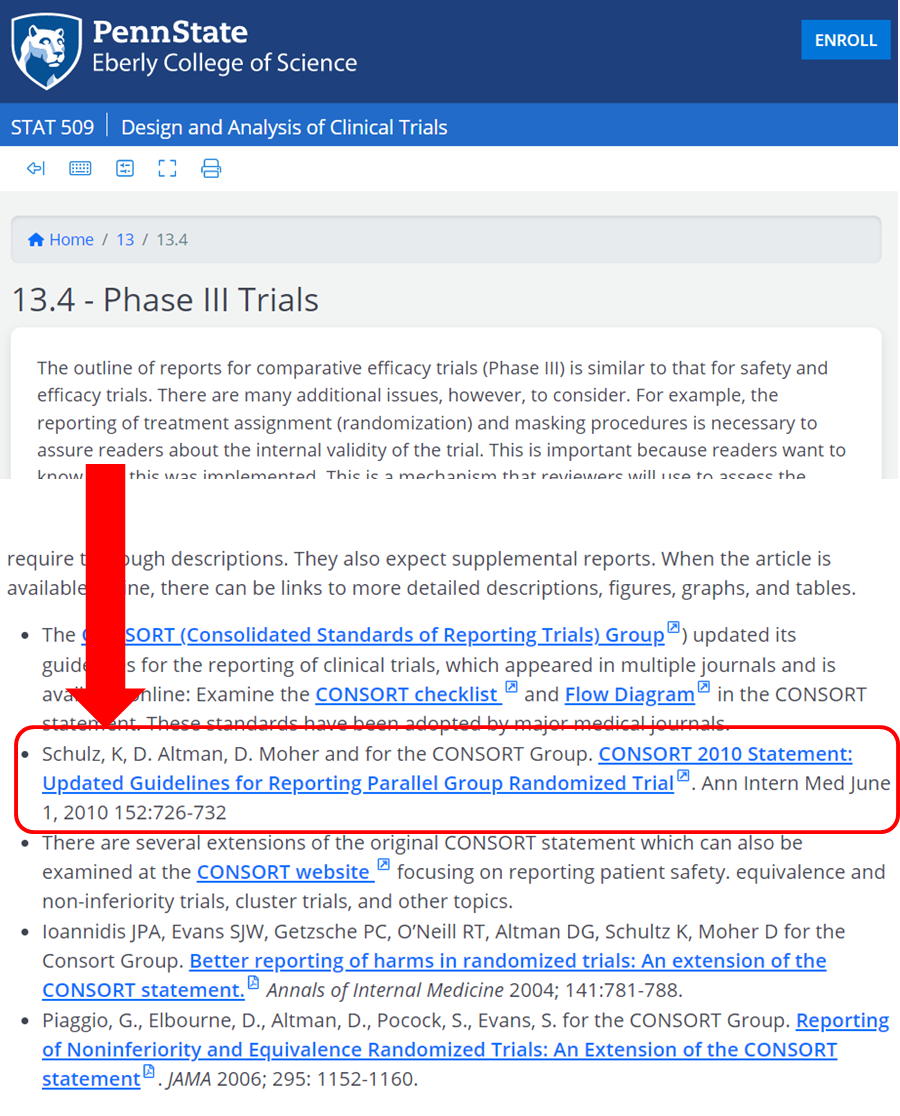


**Wiki Entry**

Reporting publication cited: G6-P03

Source URL:

<https://en.m.wikipedia.org/wiki/Blinded_experiment>

Target URL:

<https://doi.org/10.1136%2Fbmj.c332>


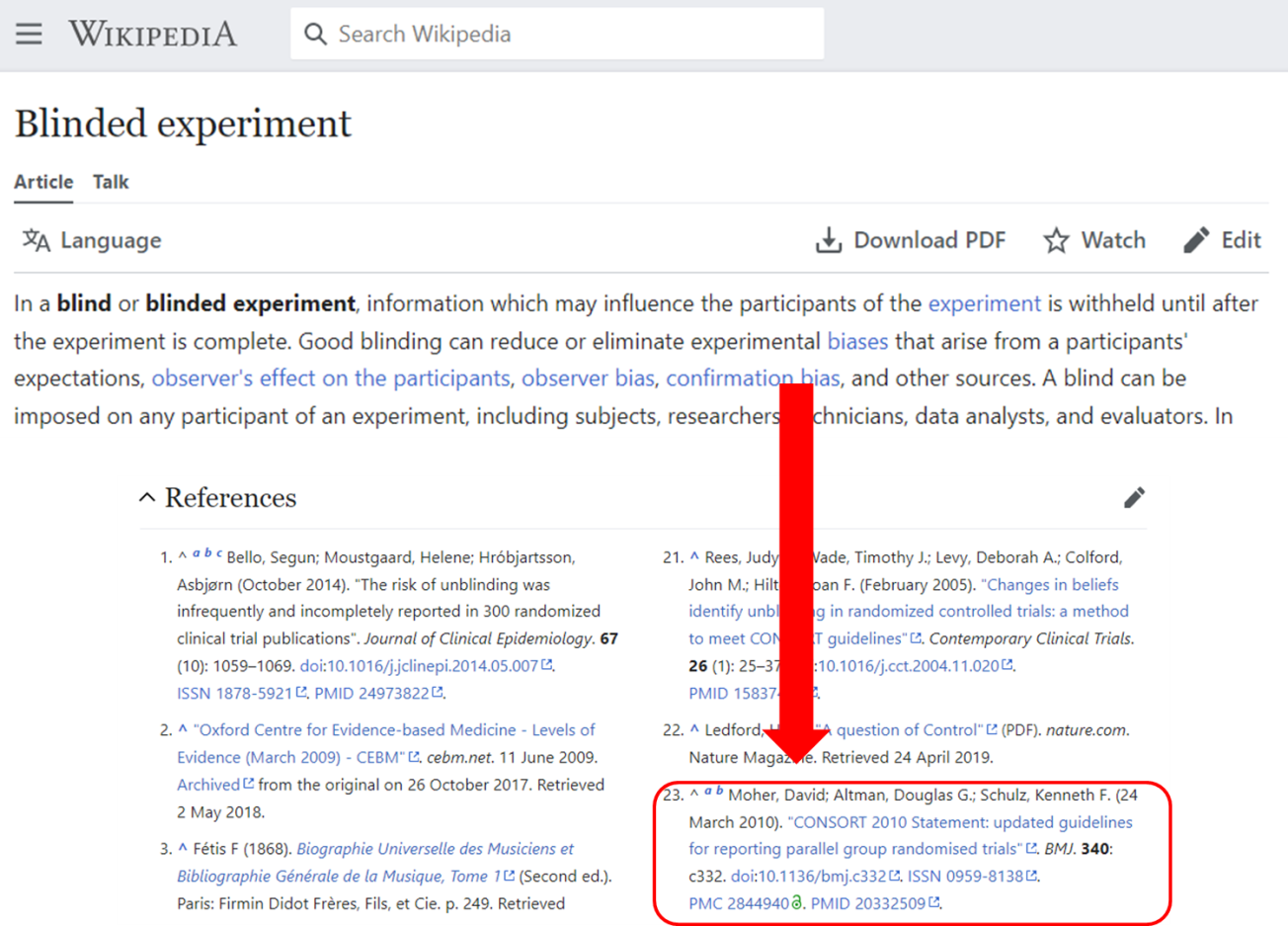

Supplement: Supplementary file 1 — Supplementary file1 (DOCX 7802 KB) [file 11192_2022_4542_MOESM1_ESM.docx]
